# Discovery of novel inhibitors disrupting HIF-1*α*/von Hippel–Lindau interaction through shape-based screening and cascade docking

**DOI:** 10.7717/peerj.2757

**Published:** 2016-12-15

**Authors:** Xin Xue, Ning-Yi Zhao, Hai-Tao Yu, Yuan Sun, Chen Kang, Qiong-Bin Huang, Hao-Peng Sun, Xiao-Long Wang, Nian-Guang Li

**Affiliations:** 1 Department of Medicinal Chemistry, Nanjing University of Chinese Medicine, Nanjing, China; 2 Department of Pharmacy, Nanjing Health-Innovating Biotechnology Co., Ltd., Nanjing, China; 3 Department of Chemistry and Biochemistry, Ohio State University, Columbus, OH, United States; 4 Division of Pharmacology, College of Pharmacy, Ohio State University, Columbus, OH, United States; 5 Department of Medicinal Chemistry, China Pharmaceutical University, Nanjing, China

**Keywords:** Protein–protein interaction, Shape-based screen, Native-docking, Cross-docking, HIF-1α/pVHL interaction

## Abstract

Major research efforts have been devoted to the discovery and development of new chemical entities that could inhibit the protein–protein interaction between HIF-1*α* and the von Hippel–Lindau protein (pVHL), which serves as the substrate recognition subunit of an E3 ligase and is regarded as a crucial drug target in cancer, chronic anemia, and ischemia. Currently there is only one class of compounds available to interdict the HIF-1*α*/pVHL interaction, urging the need to discover chemical inhibitors with more diversified structures. We report here a strategy combining shape-based virtual screening and cascade docking to identify new chemical scaffolds for the designing of novel inhibitors. Based on this strategy, nine active hits have been identified and the most active hit, 9 (ZINC13466751), showed comparable activity to pVHL with an IC50 of 2.0 ± 0.14 µM, showing the great potential of utilizing these compounds for further optimization and serving as drug candidates for the inhibition of HIF-1*α*/von Hippel–Lindau interaction.

## Introduction

Protein–protein interactions (PPIs) play a crucial role in the cellular function and form the backbones of almost all biochemical processes (Wells & McClendon, 2007). One class of PPIs with promising therapeutic potential is the interaction between the hypoxia-inducible factor 1*α* (HIF-1*α*) and the von Hippel–Lindau protein (pVHL), which acts as an essential component of a multi-subunit E3 ligase.

Hypoxia is a common pathological condition presenting in tissue tumor growth, stroke, ischemic heart disease and chronic kidney failure (Patrick et al., 1999). HIF-1*α* is a vital intermediate for the oxygen homeostasis in the cells (Muchnik & Kapplan, 2011), regulating the expression of more than 40 important target genes including vascular endothelial growth factor (VEGF), erythropoietin, glycolytic enzymes, and glucose transporters (Semenza, 1999). Under normoxic conditions, HIF-1*α* is continuously transcribed and translated. After the oxygen-dependent enzymatic hydroxylation of proline residues by prolyl hydroxylases (PHD) (Schofield & Ratcliffe, 2004), HIF-1*α* tends to be degraded through ubiquitin-proteasome system (UPS), which is normally governed by the activity of the complex consisting of the pVHL, elongins B and C, cullin 2, and ring box protein 1 (Rbx1) (Lonser et al., 2003).

Some PHD inhibitors are currently used in the clinic to stabilize HIF-1*α* (Rotili et al., 2011). However, PHD inhibitors are incapable of hydroxylating HIF-1*α*, resulting in the accumulation of HIF-1*α* and subsequently the up-regulation of the genes involved in the hypoxic response (Bagnall et al., 2014). Alternatively, peptidic inhibitors with the ability of fusing to the translocation domain and inhibiting the pVHL/HIF-1*α* interaction have been demonstrated to stabilize HIF-1*α*, confirming the idea that inhibition of pVHL/HIF-1*α* interaction can be applied as an alternative or complementary way with PHD inhibitors for the treatment of anemia (Harten, Ashcroft & Maxwell, 2010).

Starting from the minimal hydroxyproline recognition unit (Haifeng et al., 2002; Hon et al., 2002), Buckley and his coworkers reported a novel series of pyrrole derivatives to inhibit the HIF-1*α*/pVHL interaction with binding affinities at nanomolar range (Buckley et al., 2012b; Buckley et al., 2012a; Dias et al., 2014). The best ligand from their report was more potent than the model 10-mer HIF-1*α* peptide and also revealed new binding modes in the pVHL, providing an excellent starting point to validate pVHL as a drug target (Galdeano et al., 2014). However, no other chemical inhibitors with new scaffolds were reported so far, making it an intriguing idea to discover novel chemical cores and define effective hot spots in the binding pocket to understand and modulate HIF-1*α*/pVHL interactions.

**Figure 1:**
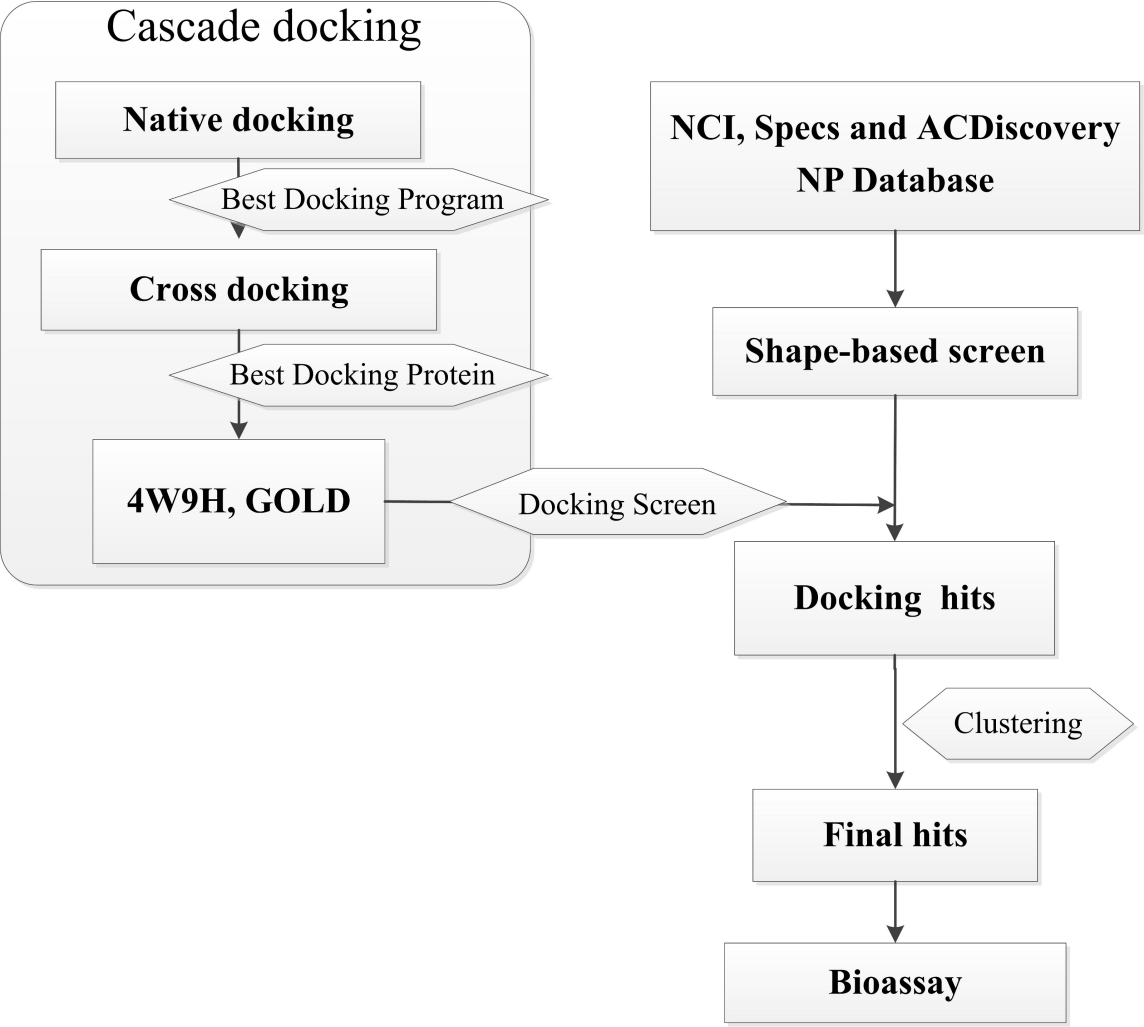
Flow diagram of the shape-based screening protocol and cascade docking procedures.

In this research, we report a computation-based method to design new inhibitors for HIF-1*α*/pVHL interactions employing shape-based modeling (Kirchmair et al., 2007; Chen et al., 2016), virtual screening and cascade docking (Xue et al., 2013) (see in Fig. 1).

## Materials and Methods

### Shape-based screening

**Figure 2:**
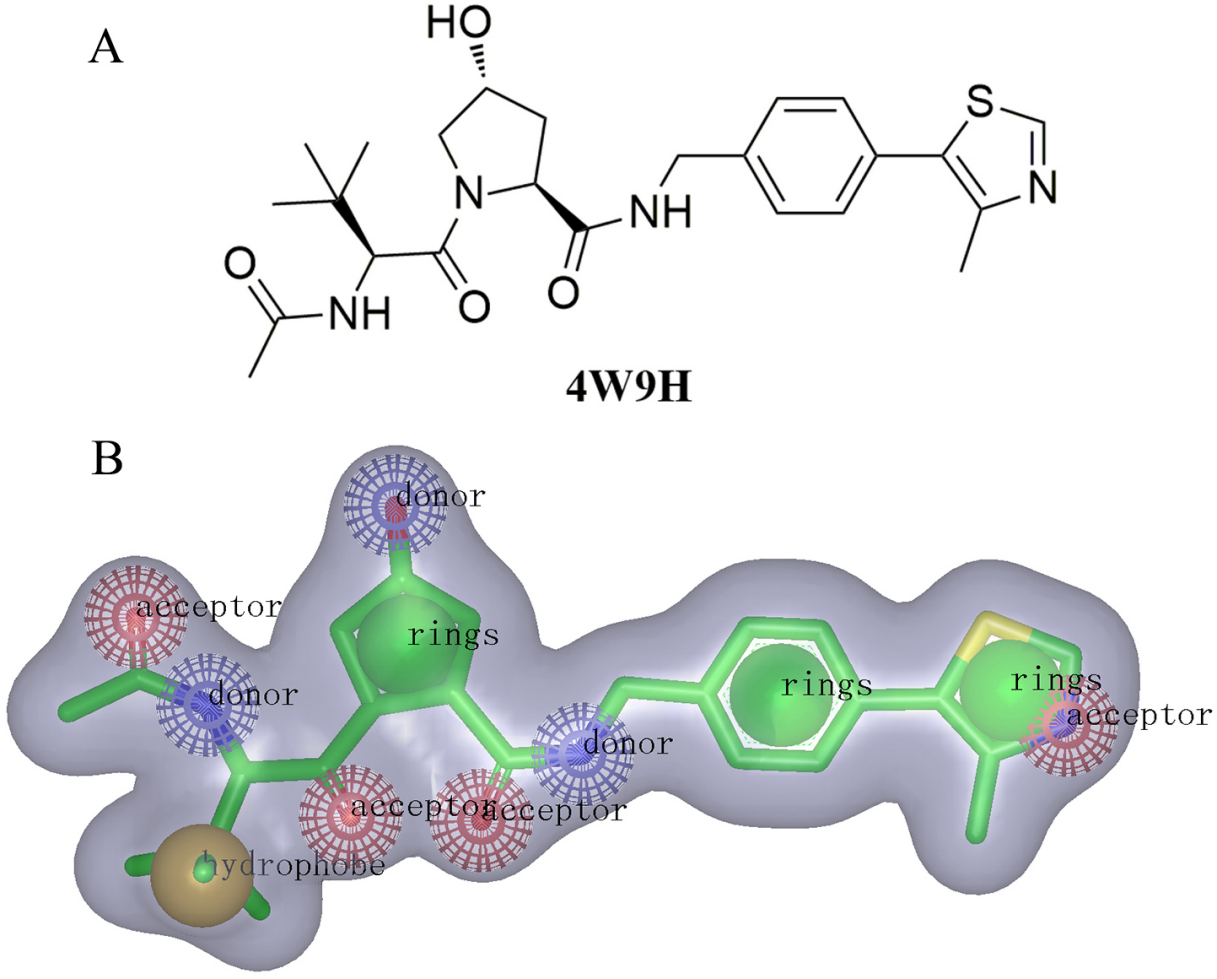
(A) The structure of the template molecule extracted from the crystal complex, PDB id **4W9H**. (B) The shape-based model where red, blue, yellow, and green balls represent hydrogen-bond acceptors, hydrogen-bond acceptor donors, hydrophobic features, and ring features.

For the discovery of new chemical structures, we utilized a strategy that combined shape-based modeling, virtual screening, and molecular docking. Rapid Overlay of Chemical Structures (ROCS) was a highly efficient shape comparison application based on the principle that molecules will form similar shape if their volumes could overlay sufficiently (Kirchmair et al., 2007; Kirchmair et al., 2009). Gaussian function (Zhou et al., 2008) was applied to calculate the molecular volume in this program. To be brief, the ROCS color force field described one molecule by the spatial arrangement of chemical features including six types: hydrogen-bond donors, hydrogen-bond acceptors, hydrophobes, anions, cations, and rings. The native ligand extracted from published X-ray co-crystal complex (PDB id: **4W9H)** with detailed interaction information between HIF-1*α* and pVHL was used as the template to generate the shape-based model (Fig. 2B). Once the model was generated, virtual screening of natural products and derivative libraries (approximately 100,000 molecules) including **NCI**, **Specs Natural Product (NP)** and **ACDiscovery NP databases** was processed for the initial screening. The combo score method, consisting of the shape Tanimoto coefficient (Godden, Xue & Bajorath, 2000; Bajusz, Racz & Heberger, 2015) and scores retrieved from the ROCS color force field, was adopted to evaluate the shape similarity between screened compounds and **4W9H** native ligand. In ROCS, the obtained combo scores ranged from 0 to 2 and the higher combo score indicated the better similarity between a given compound and the **4W9H** native ligand.

### Cascade docking

#### Native docking

The pVHL-ligand complexes crystal structures (PDB id: **3ZRC** (Buckley et al., 2012b), **3ZTC** (Van-Molle et al., 2012), **3ZTD** (Van-Molle et al., 2012), **3ZUN**, **4B9K** (Buckley et al., 2012a), **4B95** (Buckley et al., 2012a), **4BKS** (Dias et al., 2014), **4BKT** (Dias et al., 2014), **4W9C** (Galdeano et al., 2014), **4W9D** (Galdeano et al., 2014), **4W9E** (Galdeano et al., 2014), **4W9F** (Galdeano et al., 2014), **4W9G** (Galdeano et al., 2014), **4W9H** (Galdeano et al., 2014), **4W9I** (Galdeano et al., 2014), **4W9J** (Galdeano et al., 2014), **4W9K** (Galdeano et al., 2014), **4W9L** (Galdeano et al., 2014)) were employed to conduct the native docking (Verdonk et al., 2008). Those native ligands were docked back into their corresponding protein structures using GOLD (Hartshorn et al., 2007), Libdock (Diller & Merz, 2001; Rao et al., 2007) and CDOCKER  (Wu et al., 2003; Sun et al., 2011) (Discovery Studio 4.0). Structural water molecules in all receptor binding sites were retained. Hydrogens of both receptors and ligands were displayed, and the CHARMm force field (Rigby & Scott, 1983) was applied before the docking process. The default docking accuracy was applied in three programs. Ligand poses docked by GOLD, Libdock and CDOCKER were scored with Goldscore, Ligscore and Ludiscore respectively. The binding sites were defined as where the 4W9H native ligand posed. Docking results were evaluated with the root-mean-square deviation (RMSD) values to define the reliability of three docking programs. RMSD values were generated via calculating differences between the atomic distances of the docking poses and the native binding poses. The docking software that generated the smallest RMSD values would be selected to perform cross-docking.

### Cross-docking

Eighteen crystal complex structures from native-docking (Sutherland et al., 2007; Gleeson & Gleeson, 2009) were employed to perform cross docking evaluation. Native ligands were docked into all 18 complex structures using the docking software which provided the smallest RMSD value from native-docking. The docking accuracy was evaluated via calculating the RMSD difference between the reference positions of the ligand in the experimental pVHL-ligand complex and positions predicted by the docking software. The working protein structure with the smallest RMSD value was selected for further evaluation.

### Docking screening

All the molecules passing the shape-based virtual screening were aligned in GOLD, and processed with the cascade docking using the parameter discussed above. Compounds were evaluated by consensus scoring functions in GOLD with several algorithms: Ludi, Goldscore, Chemscore, LigScore1 and LigScore2. The consensus scores were calculated and ranked. The top 2% of molecules with high consensus ranking scores were retained and clustered to 10 sets based on their similarity using Tanimoto coefficient value (fingerprinter FCFP_6) (Godden, Xue & Bajorath, 2000; Bajusz, Racz & Heberger, 2015; Zhang et al., 2015) in Discovery Studio 4.0. Finally, compounds with the highest consensus scores were picked out from each set and subjected to the bioassay.

#### Fluorescence polarization binding assay

The identified compounds as potential pVHL inhibitors were purchased from **NCI**, **ACDiscovery NP** and **Specs NP** Database. For the testing of their binding affinities to pVHL protein, a sensitive and quantitative fluorescence polarization-based binding assay was performed using FAM-DEALA-Hyp-YIPD (278 nM, Biohelper Biotechnology) and V_1−213_CB (450 nM, Biohelper Biotechnology), while aqueous DEALA-Hyp-YIPD was also used as positive control. The fluorescence experiments were carried out as described in the literature (Buckley et al., 2012b). Briefly, the fluorescence polarization numbers were read on SpectraMax Paradigm Multi-mode Detection Platform (Molecular Devices) with the 485 nm excitation and 535 nm emission filters. The fluorescence intensities parallel (Intparallel) and perpendicular (Intperpedicular) to the plane of excitation were measured in black 96-well NBS assay plates (Greiner Microlon) at room temperature. The plate was then shaken for 1 min and centrifuged for 1 min. Wells containing V_1−213_CB, DMSO vehicle and FAM-DEALA-Hyp-YIPD served as maximum polarization (or minimum displacement), while wells with buffer in place of V_1−213_CB, DMSO vehicle and FAM-DEALA-Hyp-YIPD was used as minimum polarization (or maximum displacement). The inhibition percentage was determined by normalizing to maximum and minimum polarization and graphed against the log [VL]. IC_50_ values were then determined using Prism for each replicate (*n* = 9), which were then averaged to determine the mean IC_50_ and the standard error of the mean (SEM).

## Results and Discussions

The molecular shape of native ligand **4W9H** was displayed in gray shadow (Fig. 2B), which contained the hydrophobic features derived from the core of **4W9H** native ligand. The amide group acted as both the hydrogen-bond acceptor (HBA) and hydrogen-bond donor (HBD), while the hydroxy group on pyrrole could form another HBA feature. The nitrogen atom on oxazole rings was capable of forming two other HBA features. The hydrophobic feature was generally formed around the tertiary butyl group. Three nature products databases were screened against the resulted shape-based pharmacophore model to build a library for further docking screening. Subsequently, 1,000 molecules with the TanimotoCombo index larger than 0.6 were obtained to merge into a library with the highest score being 1.289.

Three factors, including the conformation of the receptor, the docking program and the scoring system, predominantly determine the quality of the docking results. Herein, native-docking was employed to define the best docking program. The crystal complex structure published on the Protein Data Bank (PDB) revealed the snapshot of the crystallization process. In consequence, a single crystal structure of the complex was able to demonstrate the best binding conformation of the flexible receptor with errors. Cross-docking (Sutherland et al., 2007; Duca, Madison & Voigt, 2008; Voigt et al., 2008) was applied as an effective method to help define the best crystal structure fitting the docking project from PDB.

According to the results from native-docking of 18 pVHL proteins structures with their native ligands (listed in Supplemental Information 1), GOLD program had yielded the smallest average RMSD (0.4211), suggesting that GOLD program was suitable for the docking of pVHL structures with other potential inhibitors. Meanwhile, the results of cross-docking (see Supplemental Information 1) also shown that docking using **4W9H** protein structure with various ligands produced the smallest average RMSD (0.4011). Combining the above results together, GOLD program and **4W9H** protein structure were selected to perform docking screening.

**Figure 3:**
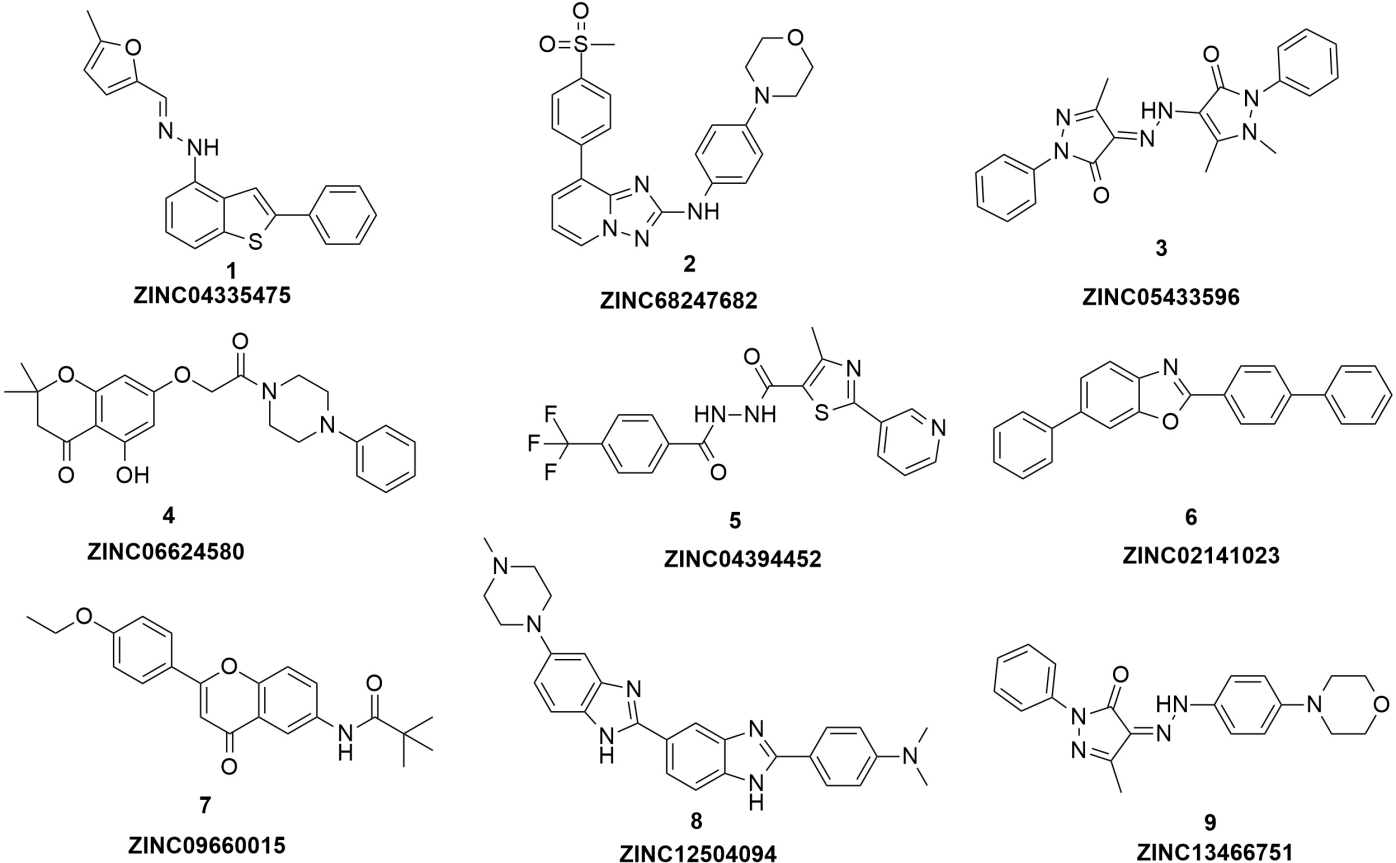
The structures of the nine hits.

**Table 1:** The inhibitory activity of the hits from virtual screening.

Compound	ZINC ID	MW	IC_50_^a^ (µM)
**1**	ZINC05433596	388	54 ± 2.31
**2**	ZINC01034728	418	58 ± 5.79
**3**	ZINC06624580	410	21 ± 1.52
**4**	ZINC04394452	406	9.0 ± 0.87
**5**	ZINC09660015	365	6.0 ± 0.63
**6**	ZINC02141023	347	102 ± 6.13
**7**	ZINC04335475	332	37 ± 1.58
**8**	ZINC12504094	451	83 ± 3.10
**9**	ZINC13466751	363	2.0 ± 0.14
**4W9H**		472	1.1 ± 0.03

**Notes.**

a The inductivity of the compound is calculated compared to the blank control, and data are presented as mean ± SEM of five separate experiments.

**Figure 4:**
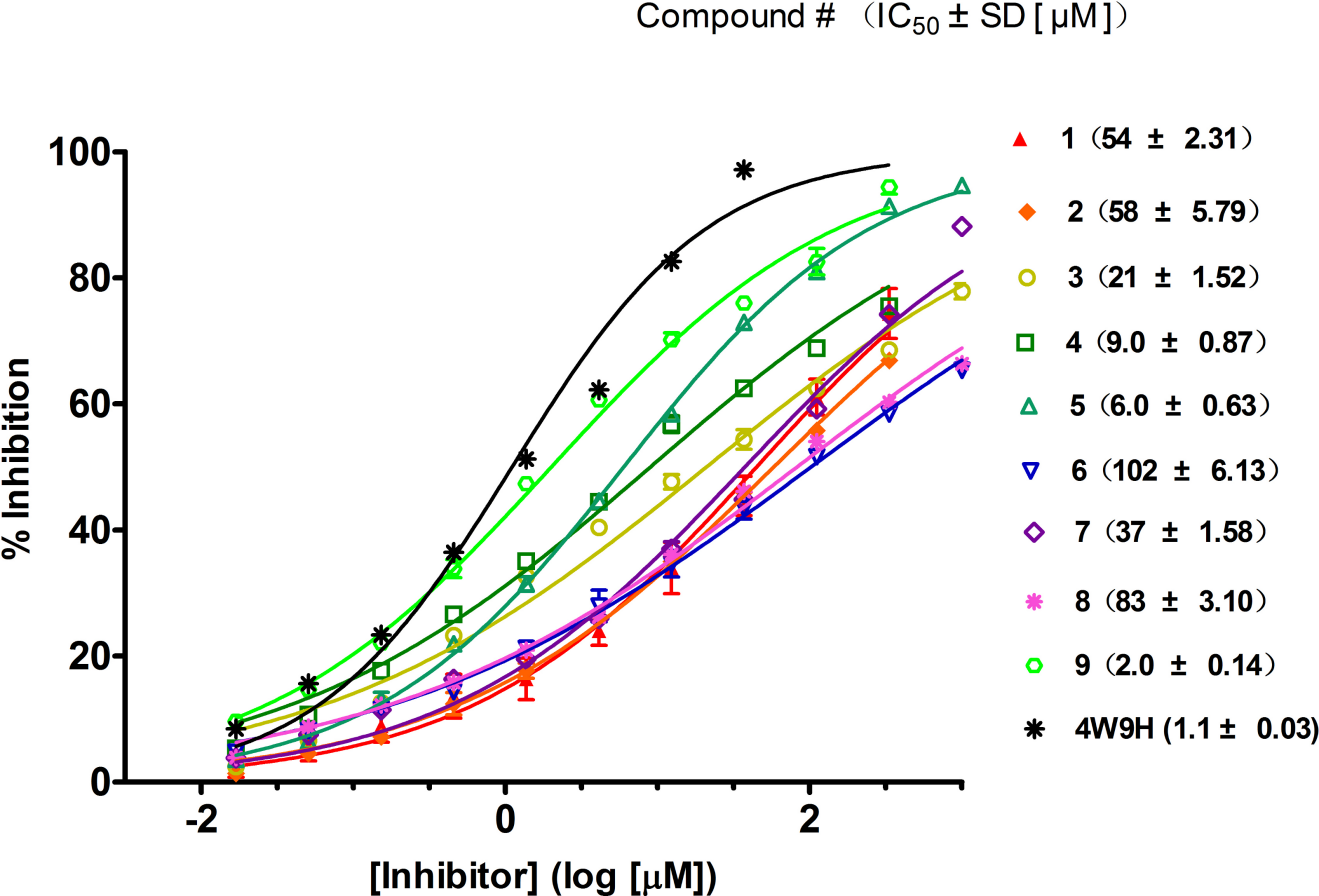
Competitive binding curves of nine hits and **4W9H** native ligand against pVHL as determined using a fluorescence-polarization-based binding assay.

Based on the docking screening results with GOLD program, 22 potential active hits with diversified chemical structures were selected and purchased for Fluorescence Polarization Binding assay. As shown in Fig. 3, nine active compounds displayed potential inhibitory activity against HIF-1*α*/pVHL protein–protein interaction, proving the effective binding affinity of the nine hits with pVHL. Most of the nine active hits exhibited moderate potency with IC_50_ value spanning from 2.0 µM to 102 µM (see Table 1 and Fig. 4). Three hits had the IC_50_ value smaller than 10 µM while only compound **6** (**ZINC02141023**) possessed the IC_50_ value larger than 100 µM. Among the nine compounds, compound** 4** (**ZINC04394452**), **5** (**ZINC09660015**) and **9** (**ZINC13466751**) showed comparable potent activity with **4W9H** native ligand whose IC_50_ value was 1.1 ± 0.03 µM. The most active compound from the pool, compound **9**, presented an IC_50_ value of 2.0 ± 0.14 µM, close enough to that of **4W9H** native ligand. The other two hits, compound **4** and **5**, also exhibited satisfactory inhibition activity of HIF-1*α*/pVHL protein–protein interaction with the IC_50_ value being 9.0 ± 0.87 µM and 6.0 ± 0.63 µM, respectively. All nine hits have smaller molecular weight (MW) than **4W9H** native ligand (MW = 472.6), indicating their potential in further structural modification possibly without losing the inhibitory activity. Among all the hits, two pyrazolone derivative compounds, compound **3** (**ZINC06624580**) and **9**, were most intriguing to us as they were similar in structure as well as potent inhibitory activity. The pyrazolone group within the two structures may be capable of generating more HBD and HBA features and inducing stronger interactions.

It was significant to note that none of these hits have been optimized yet, and there existed the possibility of improving the binding affinity of each single compound after further molecular optimization. Therefore, the discovery of the nine new chemical cores with relatively good potency was highly encouraging and could serve as promising lead compounds.

**Figure 5:**
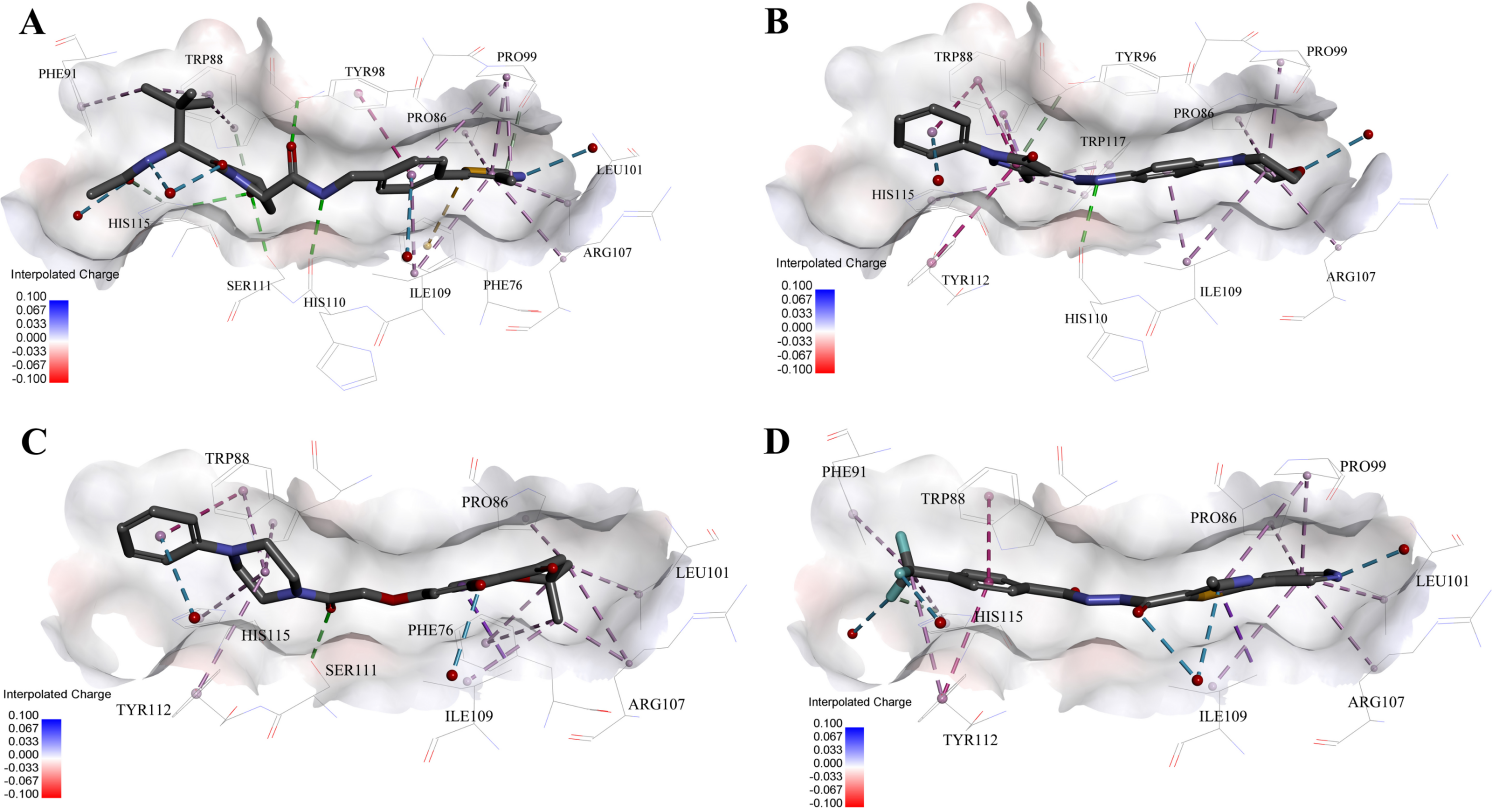
Predicted binding modes of (A) **4W9H** native ligand, (B) compound **9**, (C) compound **4** and (D) compound **5** to pVHL. The protein displayed as a gray surface of charge. Key residues close to ligands were added with labels and shown as lines. All ligands were shown with only backbone atoms using sticks. Structural water molecules were shown as red spheres. The hydrogen bonds were shown as green dash lines. The hydrophobic interactions were shown as purple dash lines.

In order to gain more detailed insight into the binding modes, all nine active hits as well as **4W9H** native ligand were docked back into the pVHL structure (PDB id: **4W9H**) with GOLD setting at default parameters for docking accuracy and scoring. As shown in Fig. 5A, four hydrogen bonds were observed between **4W9H** native ligand and His115, Ser111 and Tyr98, which ensured a correct binding pose between **4W9H** native ligand and pVHL. One *π*–*π* interaction between **4W9H** native ligand and Tyr98 together with some other hydrophobic interactions further steadied the binding between **4W9H** native ligand and pVHL.

While compound **9** possessed a comparable binding affinity with **4W9H** (see Fig. 5B), the docking results also showed an expected similar binding mode which fully occupied this pocket. In detail, compound **9** developed one hydrogen bond with His110. Another hydrogen bond was formed found between compound **9** and structural water molecules close to Pro99 which revealed that the morpholinyl group pointed outward toward the solvent environment. Compound **9** could also form two strong *π*–*π* interactions with Trp88 and one weak *π*–*π* interaction with Tyr112. The two hydrogen bonds and three *π*–*π* interactions coupled with many hydrophobic interactions greatly strengthened the binding between compound **9** and pVHL.

Two other potent compounds **4** and **5** with IC_50_ values smaller than 10 µM displayed different binding patterns with pVHL. Only one H-bond was observed between the phenolic hydroxyl of the flavone ring in compound **4** and Pro99. The binding energy was mainly contributed by other hydrophobic interactions between compound **4** and pVHL.

In contrast, no hydrogen bond was discovered in the binding mode between compound **5** and pVHL. Instead, several other hydrogen bonds were found including two bondings between fluorine atoms within trifluoromethyl group and structural water as well as another two between compound **5** and structural water. Those four hydrogen bonds revealed that compound **5** had a relatively low clogP which was often associated with a better physicochemical property. Compound **5** contacted with pVHL mainly through hydrophobic and *π*–*π* stacking interactions. Similar to **4W9H** native ligand, the benzyl fluoride of compound **5** formed two strong *π*–*π* stacking interactions with Trp88 and Tyr112. Compound **5** also had a similar molecular length with **4W9H** native ligand which may help improve the stability when contacting with pVHL.

**Figure 6:**
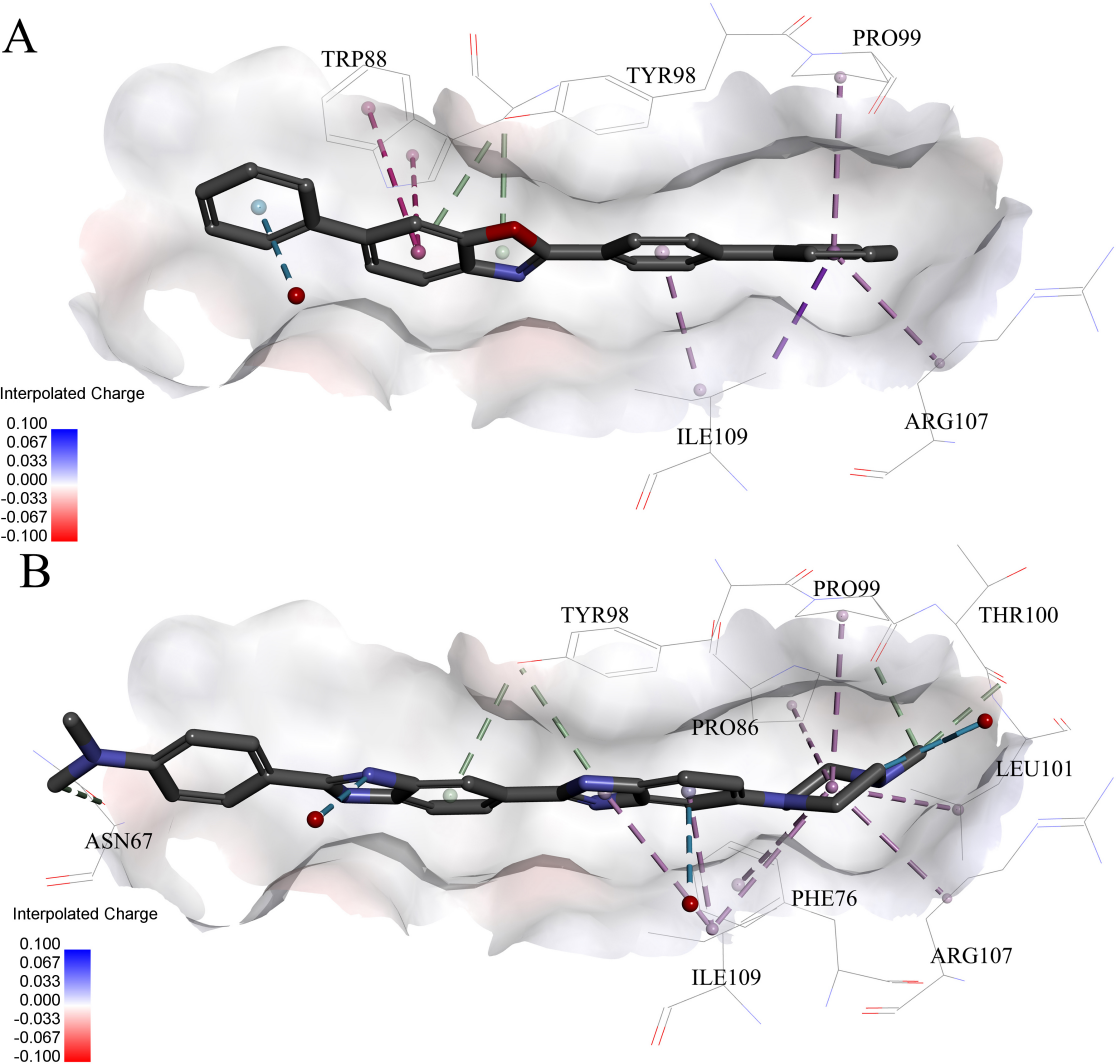
Predicted binding modes of (A) compound **6** and (B) compound **8** to pVHL. The protein displayed as a gray surface of charge. Key residues close to ligands were added with labels and shown as lines. All ligands were shown with only backbone atoms using sticks. Structural water molecules were shown as red spheres. The hydrophobic interactions were shown as purple dash lines.

Very interestingly, compound **6** and **8** (see Fig. 6), which performed worst on the docking results, demonstrated similar binding patterns with pVHL. Few interactions between compound **6** and the pVHL can be observed besides less hydrophobic interactions. Compound **6**, with a rigid and linear skeleton, kept a distance (longer than 3 Å) from the nearby amino acid residues in the binding pocket which made it difficult to form effective interactions. Hence, there was no enough force to steady the binding of compound **6** in the pocket. Similar to compound **6** both in the binding mode and the molecular rigidity, compound **8** hardly formed any interactions besides the weak *π*–*π* stacking interaction between its aromatic ring and Phe91. However, the binding affinity of compound **8** was slightly better than compound **6** possibly because that the nitrogen atoms in compound **8** can supply more polar interactions with pVHL. Binding modes of the other compounds, **1**, **3** and **7**, were included in Supplemental Information 1.

## Conclusions

In conclusion, our report has demonstrated a great effort in the optimization of the virtual screening (VS) strategy for protein–protein interactions. Comparing to the classic VS strategy for HIF-1*α*/pVHL protein–protein interaction, the strategy presented here combined both shape-based pharmacophore model and cascade docking based on the idea that molecules with similar shape tend to possess similar activities. With the assistance of ROCS, a shape-based pharmacophore model derived from the native ligand of the crystal structure of pVHL (PDB id: **4W9H**) was successfully constructed which revealed distinct interactions between inhibitors and pVHL. Shape-based screening, as well as native and cross docking, were employed to help identify active compounds ranked by the shape Tanimoto coefficient.

Subsequently, nine compounds with novel skeletons were discovered to be capable of inhibiting the HIF-1*α*/pVHL protein–protein interaction with micromolar inhibiting activity by fluorescence polarization binding assay. The best compound **9** among those nine hits had a potent binding affinity to pVHL similar to the positive control **4W9H** native ligand. The docking results also emphasized the importance of the *π*–*π* stacking interaction formed by Trp88, His115 or Tyr98 in the binding pocket that could maintain the binding between the receptor and inhibitors. In addition, our work has proved that the molecular flexibility was a key factor for the inhibitors to occupy the binding pocket fully and avoided crash with protein surface. Considering the relative simple structures and low molecular weight, the identified nine hits here could provide decent chemical cores in designing novel HIF-1*α*/pVHL protein–protein interaction inhibitors with high ligand efficiency.

##  Supplemental Information

10.7717/peerj.2757/supp-1 Supplemental Information 1 Raw data of the materials for docking experiments, shape-based modeling, the results of Native-Docking and Cross-Docking, the screening scores of GOLD docking, the inhibitory activities and the predicted binding modes

10.7717/peerj.2757/supp-2 Supplemental Information 2 Raw data of the results of nine compounds, 3ZRC native ligand and 4W9H native ligand docked with pVHL exported from the GOLD program

10.7717/peerj.2757/supp-3 Supplemental Information 3 Raw data exported from the SpectraMax Paradigm Multi-mode Detection Platform (Molecular Devices) applied for data analyses and preparation for Table 1, Fig. 4 and Supporting Figure 4, 7, 10, 13, 16, 19, 22, 25, 28 and 31

## Additional Information and Declarations

### Data Availability

The following information was supplied regarding data availability: The raw data has been supplied as Supplemental Files.
