# Supplementary material for: Discovery of novel inhibitors disrupting HIF-1α/von Hippel–Lindau interaction through shape-based screening and cascade docking
Source: PeerJ. 2016 Dec 15;4:e2757. doi: 10.7717/peerj.2757 (PMC5162400; doi:10.7717/peerj.2757)
Supplement: Supplemental Information 1 [file peerj-04-2757-s001.docx]

**Discovery of novel inhibitors disrupting HIF-1α/von Hippel−Lindau interaction through shape-based screening and cascade docking**

*Xin Xue,* *^a^ Ning-Yi Zhao, ^b^* Hai-Tao Yu, *^a^* *Yuan Sun, ^c^* *Chen Kang,* *^d^* *Qiong-Bin Huang,^a^ Hao-Peng Sun, ^e^ Xiao-Long Wang* *^a,*^ Nian-Guang Li*, *^a,*^*

1. The information about 18 crystal complex and their native ligands for virtual screening.

**Supporting Table 1** The crystal complex and their native ligands for virtual screening.

| **PDB ID** | **Resolution** | Sequence | MW of native ligands | *K*_d_ of native ligand(μM) |
| --- | --- | --- | --- | --- |
| **3ZRC** | 2.9Å | LEU63-GLU204 | 410 | 5.3 |
| **3ZTC** | 2.65 Å | LEU63-GLU204 | 419 | 27.7 |
| **3ZTD** | 2.79 Å | LEU63-GLU204 | 401 | 96.2 |
| **3ZUN** | 2.5 Å | VAL62-GLU204 | 390 | To Be Published |
| **4B9K** | 2.0 Å | VAL62-THR202 | 451 | 0.7 |
| **4B95** | 2.8 Å | VAL62-GLN203 | 393 | 52(IC_50_) |
| **4BKS** | 2.2 Å | VAL62-THR202 | 329 | 150 |
| **4BKT** | 2.35 Å | VAL62-LEU201 | 267 | 240 |
| **4W9C** | 2.2 Å | VAL62-THR202 | 385 | 22.2 |
| **4W9D** | 2.2 Å | VAL62-THR202 | 399 | 10.2 |
| **4W9E** | 2.6 Å | VAL62-THR202 | 402 | 7.1 |
| **4W9F** | 2.1 Å | VAL62-GLN203 | 416 | 3.3 |
| **4W9G** | 2.7 Å | VAL62-THR202 | 416 | 6.5 |
| **4W9H** | 2.1 Å | VAL62-THR202 | 473 | 0.2 |
| **4W9I** | 2.4 Å | VAL62-THR202 | 473 | 1.1 |
| **4W9J** | 2.2 Å | VAL62-THR202 | 586 | 0.6 |
| **4W9K** | 2.1 Å | VAL62-THR202 | 620 | 0.3 |
| **4W9L** | 2.2 Å | VAL62-THR202 | 586 | 9.5 |


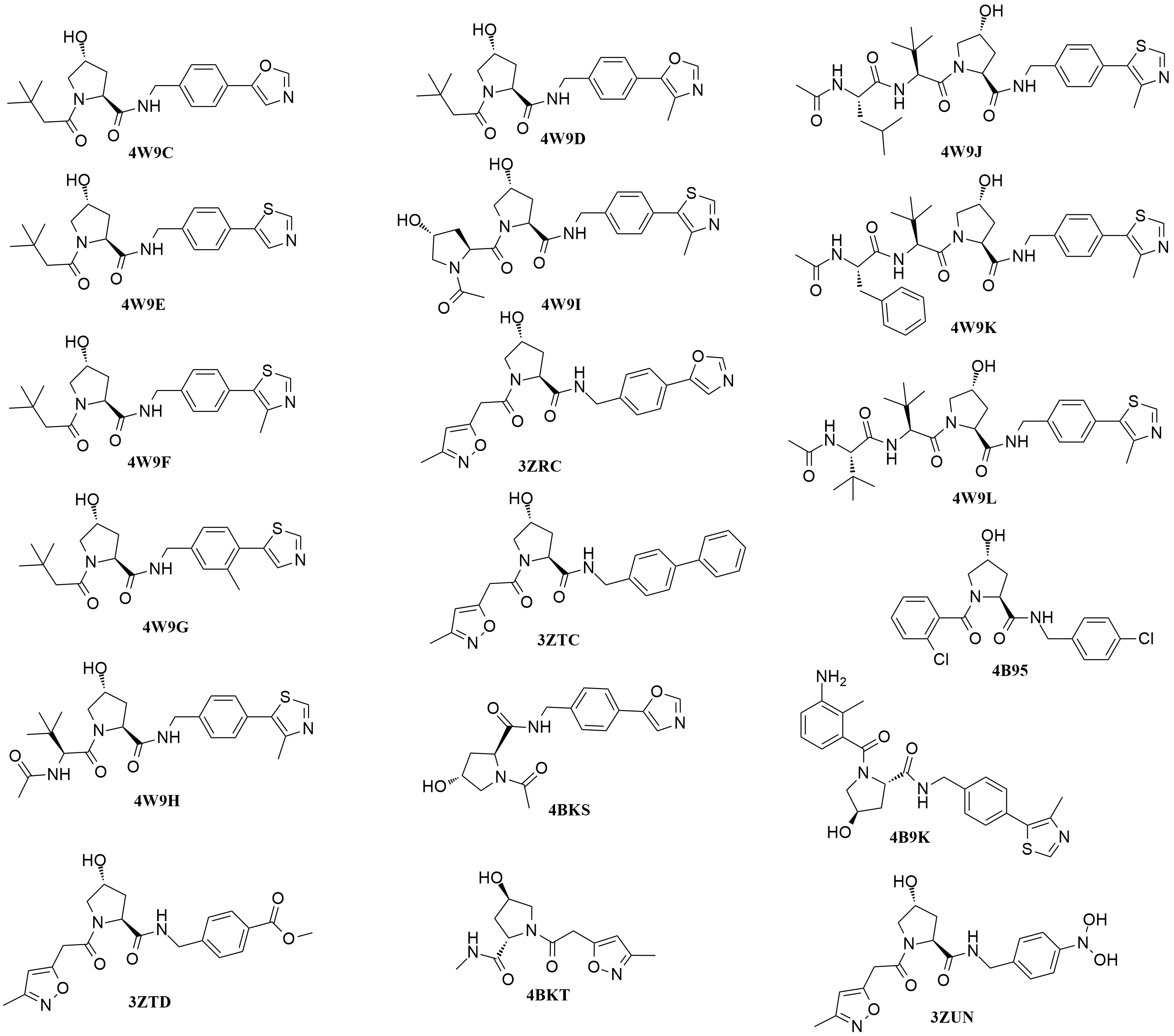


**Supporting Figure 1** The structure of the eighteen native ligands.

1. Shape-based modeling


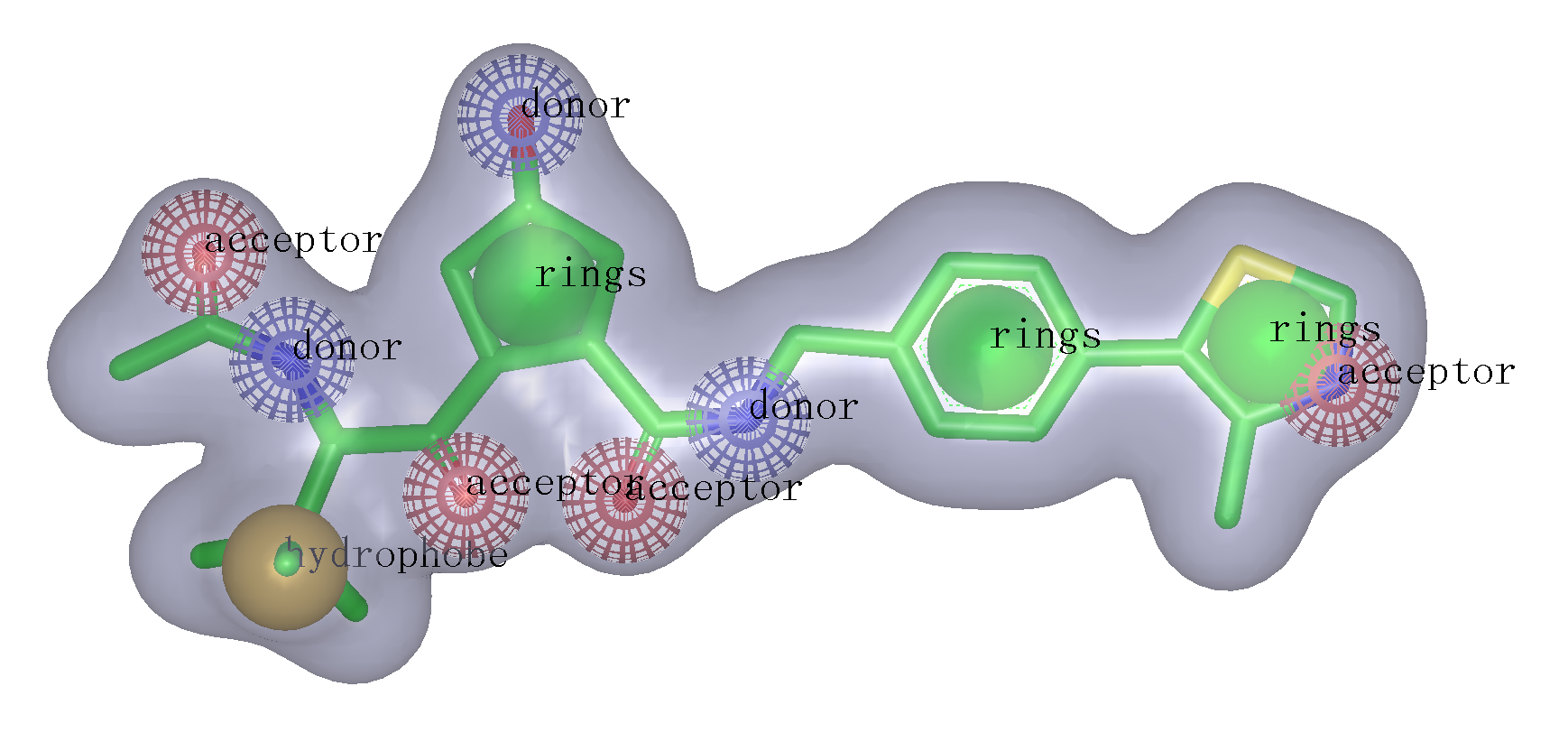


**Supporting Figure 2** The shape-based model constructed from the native ligand of **3ZRC**, in which red, blue and green ball represent hydrogen-bond acceptor, donor and ring feature.

1. Native-Docking

The pVHL-ligand complexes (PDB Code：**3ZRC**, **3ZTC**, **3ZTD**, **3ZUN**, **4B9K**, **4B95**, **4BKS**, **4BKT**, **4W9C**, **4W9D**, **4W9E**, **4W9F**, **4W9G**, **4W9H**, **4W9I**, **4W9J**, **4W9K**, **4W9L**) were used to conduct Native-Docking. These ligands were docked back into their corresponding protein structures using GOLD, Libdock and CDOCKER (DS4.0). The docking results were evaluated through comparison of the best docked ligands binding modes with the experimental ones. The root-mean-square deviation (RMSD) was used to compare differences between the atomic distances of the docked poses and the real co-crystallized pose to measure docking reliability. The docking software with the smallest RMSD would be selected to perform Cross-Docking.

**Supporting Table2** The native ligand from virtual screening.

| Docking software | | | |
| --- | --- | --- | --- |
| **PDB Code** | Libdock | CDOCKER | GOLD |
| **3ZRC** | 0.3979 | 1.5859 | 0.2413 |
| **3ZTC** | 1.1709 | 2.0197 | 0.2296 |
| **3ZTD** | 2.9709 | 0.9629 | 1.4900 |
| **3ZUN** | 8.6443 | 0.7471 | 0.2741 |
| **4B9K** | 1.8186 | 0.2671 | 0.1600 |
| **4B95** | 1.0947 | 1.1892 | 0.2580 |
| **4BKS** | 6.3324 | 1.5518 | 0.4290 |
| **4BKT** | 2.3457 | 0.5720 | 0.2156 |
| **4W9C** | 1.6034 | 0.9084 | 0.1562 |
| **4W9D** | 1.7468 | 0.3214 | 0.2032 |
| **4W9E** | 1.7304 | 0.3466 | 0.1851 |
| **4W9F** | 1.9646 | 1.4870 | 0.2004 |
| **4W9G** | 1.1303 | 1.2045 | 0.1547 |
| **4W9H** | 1.4795 | 0.3736 | 0.6202 |
| **4W9I** | 2.0986 | 0.4459 | 0.2173 |
| **4W9J** | 2.3550 | 0.4582 | 0.9678 |
| **4W9K** | 6.7849 | 1.6250 | 1.0809 |
| **4W9L** | 2.0833 | 1.8496 | 0.4970 |
| Av *^a^* | 2.6529 | 0.9953 | 0.4211 |

^a^Average RMSD values of native ligand poses referring to their native poses.

1. Cross-Docking

Eighteen complexes used in Native-Docking were used to perform Cross-Docking evaluation. The native ligands were docked into all complex structures using the docking software confirmed by Native-Docking. The docking reliability was evaluated by calculating the RMSD difference of each ligand between the reference positions of the ligand in the experimental pVHL-ligand complex and positions predicted by the docking software. Finally the working protein structure was selected which had the smallest RMSD.

**Supporting Table 3** The eighteen native ligands for Cross-Docking.

| **PDB Code** | 3ZRC Ligand | 3ZTC Ligand | 3ZTD Ligand | 3ZUN Ligand | 4B9K Ligand | 4B95 Ligand | 4BKS Ligand | 4BKT Ligand | 4W9C Ligand | 4W9D Ligand | 4W9E Ligand | 4W9F Ligand | 4W9G Ligand | 4W9H Ligand | 4W9I Ligand | 4W9J Ligand | 4W9K Ligand | 4W9L Ligand | Av*^a^* |
| --- | --- | --- | --- | --- | --- | --- | --- | --- | --- | --- | --- | --- | --- | --- | --- | --- | --- | --- | --- |
| **3ZRC** | 0.2413 | 0.6629 | 0.3528 | 0.0953 | 1.8848 | 1.4381 | 1.2900 | 7.3605 | 0.1601 | 0.0951 | 0.2809 | 0.1022 | 1.1659 | 0.4628 | 0.5922 | 0.8218 | 1.2110 | 0.3208 | 1.0299 |
| **3ZTC** | 0.1524 | 0.2296 | 0.3528 | 0.8763 | 0.1923 | 0.2781 | 3.7962 | 0.4329 | 0.3766 | 1.2640 | 0.0614 | 0.1333 | 1.0627 | 0.2834 | 1.1700 | 0.4568 | 0.4438 | 0.5697 | 0.6740 |
| **3ZTD** | 0.1460 | 0.9008 | 1.4900 | 0.8186 | 0.2906 | 0.3322 | 2.6000 | 0.8369 | 0.9430 | 1.1583 | 1.3389 | 0.3888 | 1.3653 | 0.8936 | 0.1672 | 3.4068 | 0.9258 | 0.8335 | 1.0465 |
| **3ZUN** | 1.3827 | 0.7243 | 1.5601 | 0.2741 | 1.4231 | 0.9821 | 2.5961 | 0.1947 | 0.4197 | 3.2000 | 0.3509 | 3.1894 | 1.1986 | 3.6205 | 0.6334 | 1.0909 | 1.9519 | 1.0642 | 1.4365 |
| **4B9K** | 0.1654 | 1.0368 | 0.3903 | 1.0723 | 0.1600 | 0.2157 | 2.6715 | 3.2135 | 0.1463 | 0.2634 | 1.0777 | 0.3241 | 0.0773 | 0.1497 | 0.8828 | 1.1266 | 1.6818 | 0.5881 | 0.8469 |
| **4B95** | 1.0423 | 0.4173 | 0.5831 | 0.9207 | 0.1657 | 0.2580 | 2.5636 | 0.1497 | 0.3443 | 2.3122 | 1.1585 | 1.0484 | 1.1954 | 0.5835 | 1.2166 | 10.2217 | 1.1246 | 1.4183 | 1.4847 |
| **4BKS** | 0.2843 | 0.9713 | 0.1870 | 0.8611 | 0.0927 | 1.9351 | 0.4290 | 3.0368 | 0.2102 | 1.1663 | 0.2762 | 0.9860 | 0.2529 | 0.4533 | 0.2249 | 1.1480 | 0.2484 | 2.2006 | 0.8313 |
| **4BKT** | 0.4975 | 0.3182 | 0.9019 | 0.4010 | 0.4516 | 1.1074 | 3.9295 | 0.2116 | 8.4752 | 5.0226 | 0.3510 | 1.8536 | 9.3330 | 3.7268 | 3.9823 | 3.6449 | 10.6145 | 3.4946 | 3.2398 |
| **4W9C** | 0.3517 | 0.8259 | 0.1638 | 0.3112 | 0.5411 | 0.5072 | 3.6609 | 0.2939 | 0.1562 | 0.4467 | 0.2106 | 1.0513 | 0.1216 | 0.3488 | 0.2330 | 1.1845 | 2.5355 | 0.5376 | 0.7490 |
| **4W9D** | 0.2120 | 0.7185 | 1.0039 | 0.9646 | 0.1601 | 1.5671 | 0.9231 | 7.8430 | 0.9726 | 0.2032 | 0.1323 | 1.0780 | 0.2786 | 0.2286 | 0.1078 | 2.0448 | 10.8870 | 2.1018 | 1.7459 |
| **4W9E** | 1.0114 | 0.9638 | 0.9406 | 0.8512 | 2.4250 | 0.0853 | 3.3523 | 0.2147 | 0.3139 | 0.3445 | 0.1851 | 1.0888 | 0.2021 | 0.2114 | 1.2239 | 0.5723 | 0.4309 | 0.8755 | 0.8496 |
| **4W9F** | 0.0525 | 0.7593 | 0.8455 | 0.2683 | 0.3225 | 0.2017 | 0.5939 | 0.0719 | 1.1400 | 1.2636 | 0.1505 | 0.2004 | 0.2207 | 0.2014 | 1.0649 | 2.0494 | 0.7959 | 0.4098 | 0.5896 |
| **4W9G** | 0.9974 | 0.2159 | 0.2958 | 0.1477 | 0.4107 | 0.5607 | 7.6049 | 2.8800 | 0.1600 | 0.6113 | 1.1643 | 0.1656 | 0.1547 | 0.6994 | 1.1672 | 1.2568 | 0.8910 | 1.4204 | 1.1558 |
| **4W9H** | 0.2586 | 0.3438 | 1.0958 | 0.2245 | 0.1396 | 0.1576 | 1.3445 | 0.1112 | 0.3592 | 0.1971 | 0.1883 | 0.3398 | 0.2652 | 0.6202 | 0.0874 | 0.6032 | 0.6526 | 0.2308 | 0.4011 |
| **4W9I** | 0.4169 | 0.3868 | 0.7519 | 0.2553 | 0.1778 | 0.2369 | 1.5265 | 0.1604 | 0.3003 | 0.1427 | 0.1542 | 1.2118 | 1.2611 | 0.5984 | 0.2174 | 0.7570 | 0.4422 | 0.6941 | 0.5384 |
| **4W9J** | 0.1155 | 0.9300 | 0.1433 | 0.9026 | 1.5389 | 1.5758 | 0.9230 | 0.2056 | 0.2900 | 1.0983 | 0.1714 | 0.4252 | 0.2660 | 0.4048 | 0.6151 | 0.9678 | 0.5977 | 0.6376 | 0.6560 |
| **4W9K** | 1.0762 | 0.9006 | 1.1827 | 1.4786 | 2.7473 | 0.2844 | 0.7888 | 0.2360 | 1.0097 | 0.2428 | 0.8734 | 1.0709 | 0.3842 | 0.2697 | 0.3158 | 0.5433 | 1.0809 | 0.3991 | 0.8269 |
| **4W9L** | 1.0869 | 0.1656 | 0.5050 | 0.2124 | 0.1564 | 1.4958 | 1.0601 | 0.0939 | 0.2535 | 1.0471 | 0.8460 | 1.1323 | 0.1707 | 0.1668 | 0.0479 | 0.7258 | 1.5493 | 0.4970 | 0.6229 |

^a^Average RMSD values of native ligand poses referring to their native poses.

1. The ZINC numbers, the shape scores generated by ROCS and the docking scores of the 22 hits screened by GOLD.

**Supporting Table 4** The screening scores of the 22 hits retained after GOLD docking.

| **NAME** | **MW** | **COMBO SCORE** | **shape Tanimoto** | **-CDocker interaction ENERGY** | **LibDock Score** | **GOLD Score Fitness** |
| --- | --- | --- | --- | --- | --- | --- |
| **ZINC72320666** | 408.53 | 1.063 | 0.687 | 33.534 | 132.79 | 38.3046 |
| **ZINC01034728** | 418.491 | 1.033 | 0.67 | 34.2441 | 122.031 | 69.7957 |
| **ZINC02141023** | 347.409 | 0.977 | 0.757 | 30.6748 | 115.831 | 53.0827 |
| **ZINC30879352** | 377.498 | 1.076 | 0.584 | 29.1872 | 137.681 | 51.3743 |
| **ZINC13466751** | 363.413 | 1.026 | 0.695 | 37.7583 | 129.798 | 45.0739 |
| **ZINC22936870** | 463.595 | 1.039 | 0.762 | 41.583 | 139.936 | 30.2952 |
| **ZINC08879609** | 445.421 | 1.096 | 0.659 | 41.5329 | 145.91 | 54.779 |
| **ZINC30879372** | 378.463 | 1.243 | 0.726 | 32.9687 | 135.286 | 55.2002 |
| **ZINC12504094** | 452.574 | 1.045 | 0.76 | 42.3023 | 124.139 | 34.8237 |
| **ZINC00150576** | 332.313 | 0.975 | 0.766 | 29.3417 | 118.105 | 49.3516 |
| **ZINC30879611** | 425.521 | 1.042 | 0.739 | 37.8785 | 126.458 | 18.419 |
| **ZINC03125768** | 487.363 | 1.066 | 0.685 | 26.11 | 114.014 | 50.7593 |
| **ZINC04335475** | 334.395 | 1.02 | 0.693 | 27.9074 | 106.228 | 50.1269 |
| **ZINC49169623** | 346.383 | 0.968 | 0.663 | 35.5026 | 130.224 | 62.7553 |
| **ZINC09660015** | 365.422 | 0.949 | 0.679 | 37.4662 | 117.407 | 41.0432 |
| **ZINC04394452** | 406.382 | 0.92 | 0.619 | 33.6854 | 124.592 | 42.0553 |
| **ZINC02413444** | 457.303 | 0.892 | 0.71 | 43.9495 | 133.06 | 59.9172 |
| **ZINC30879401** | 392.512 | 1.053 | 0.562 | 36.5012 | 139.876 | 45.12 |
| **ZINC02151712** | 398.45 | 0.908 | 0.68 | 36.4656 | 134.639 | 48.8735 |
| **ZINC20224030** | 402.415 | 0.901 | 0.67 | 32.3553 | 114.18 | 41.6924 |
| **ZINC09659909** | 385.412 | 0.995 | 0.72 | 34.9939 | 123.223 | 42.7912 |
| **ZINC05433596** | 388.423 | 1.002 | 0.667 | 40.1133 | 129.01 | 46.6692 |

1. The chemical structures, the curve of inhibitory and the binding modes of the nine hits and **4W9H**.





**Supporting Figure 3** the structure of **4W9H**


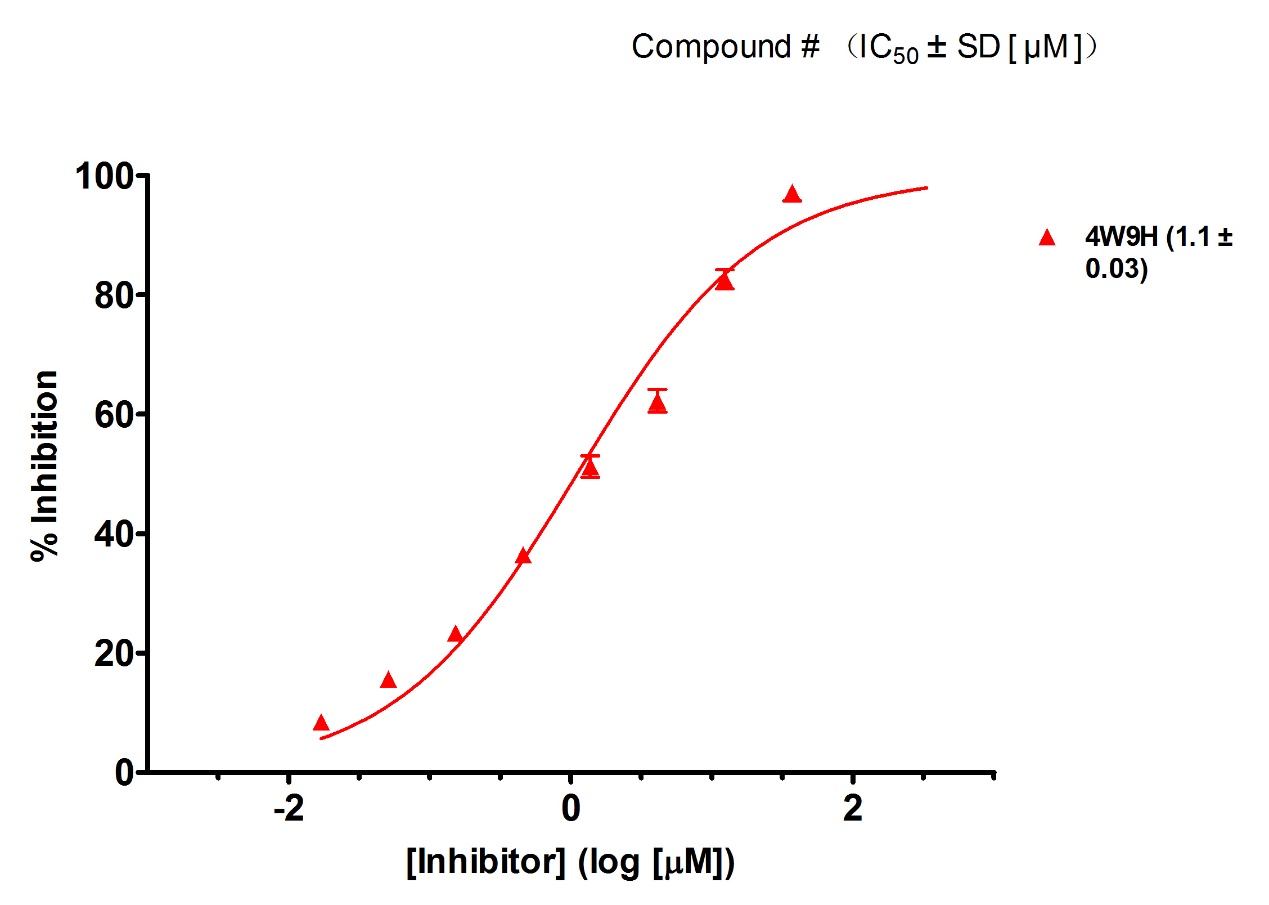


**Supporting Figure 4** the curve of inhibitory of **4W9H**


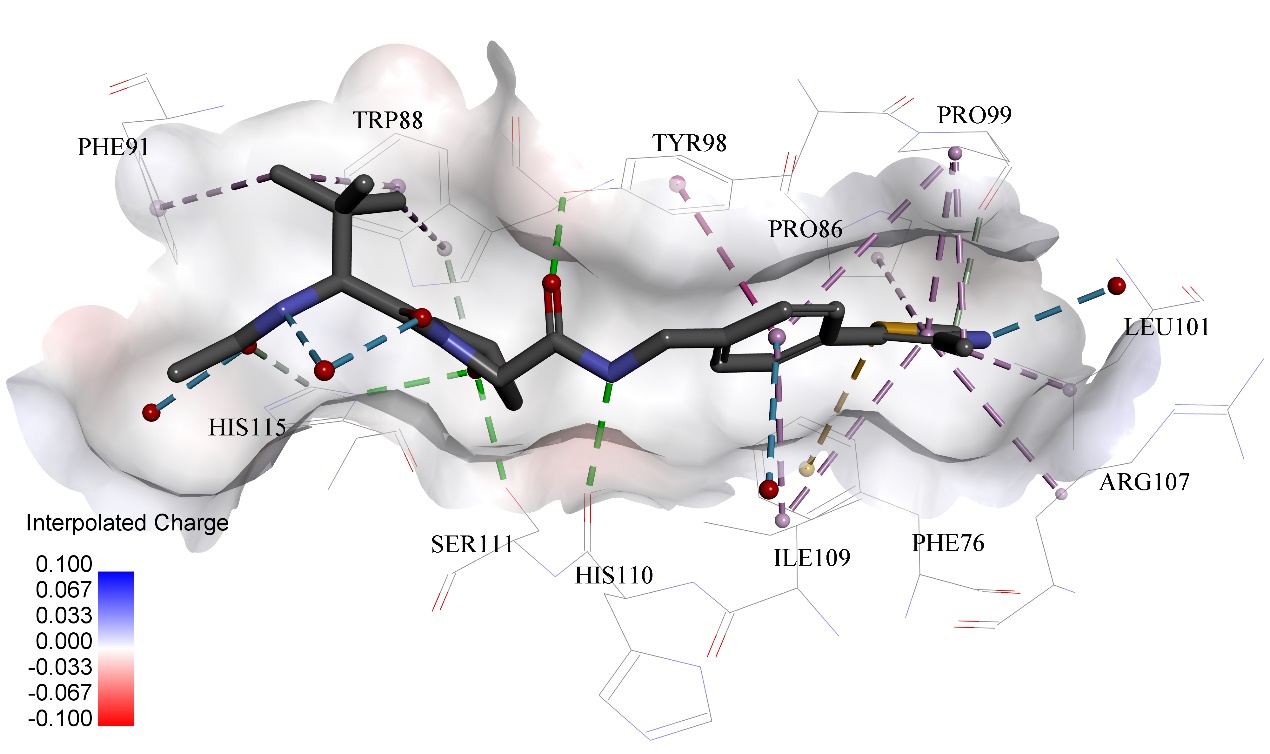


**Supporting Figure 5** Predicted binding modes of compound **4W9H** to pVHL. The protein displayed as a gray surface and its key residues were shown with labels. All compounds are shown with only backbone atoms.





**Supporting Figure 6** the structure of **1**


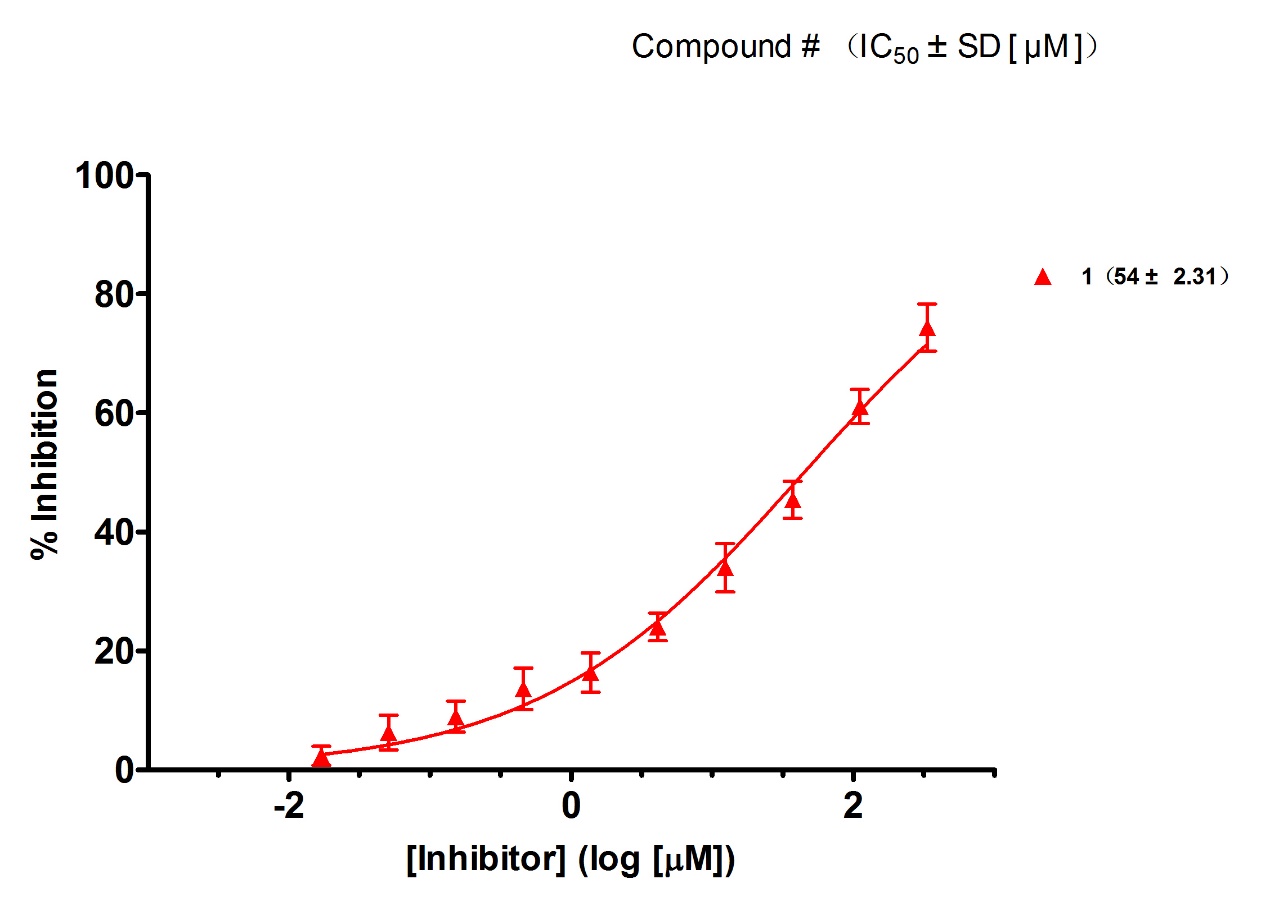


**Supporting Figure 7** the curve of inhibitory of **1**


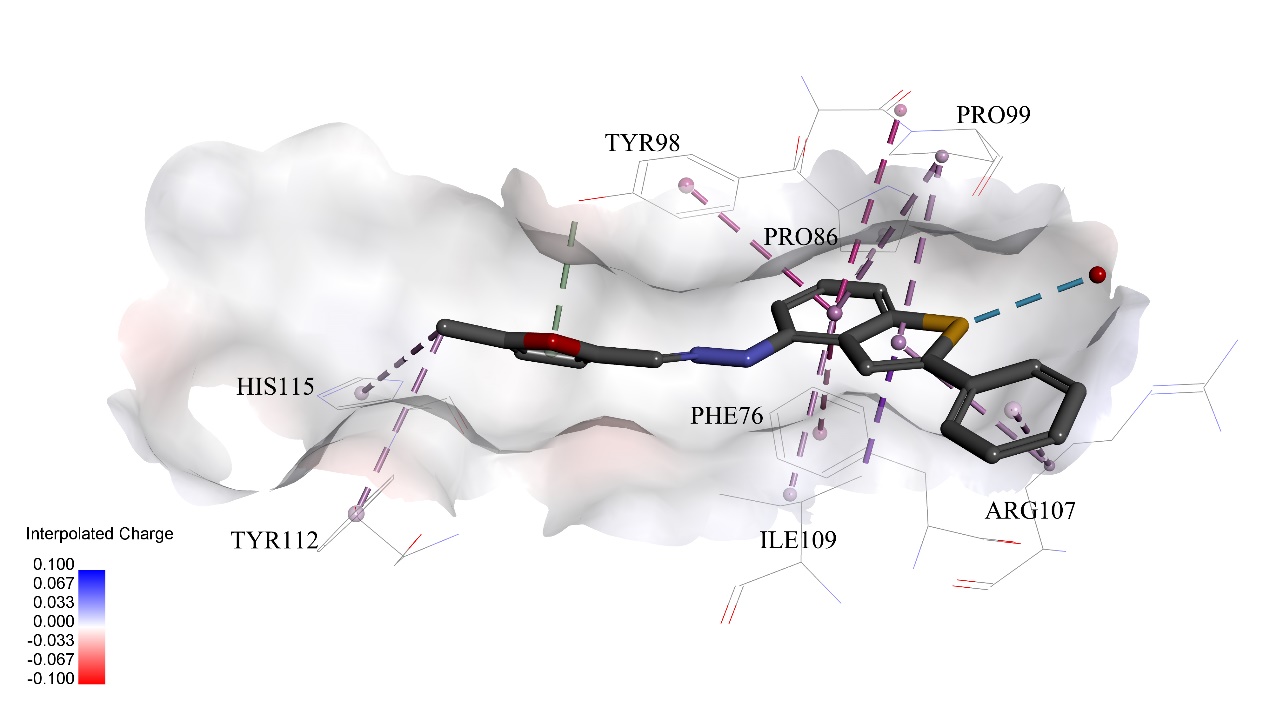


**Supporting Figure 8** Predicted binding modes of compound **1** to pVHL. The protein displayed as a gray surface and its key residues were shown with labels. All compounds are shown with only backbone atoms.





**Supporting Figure 9** the structure of **2**


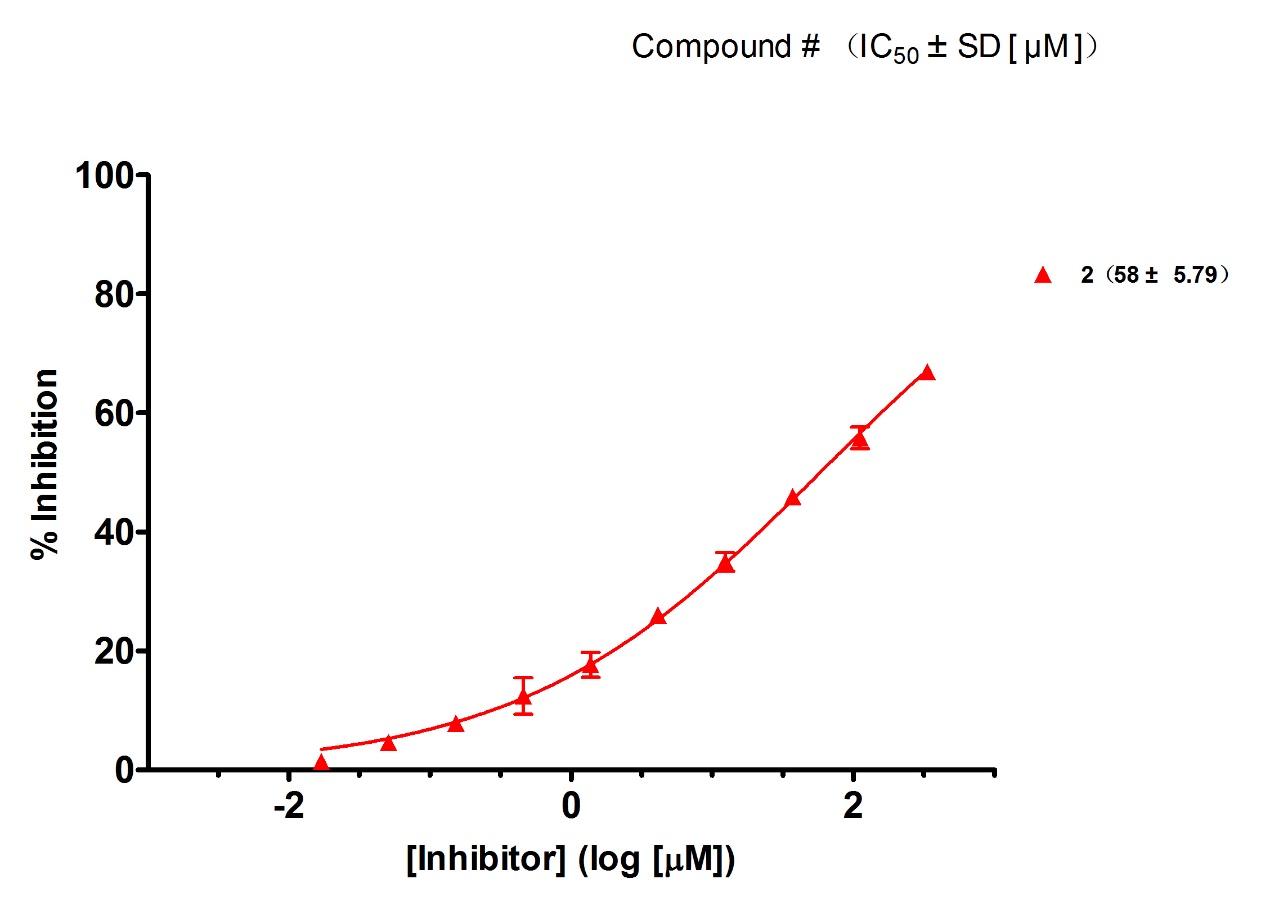


**Supporting Figure 10** the curve of inhibitory of **2**


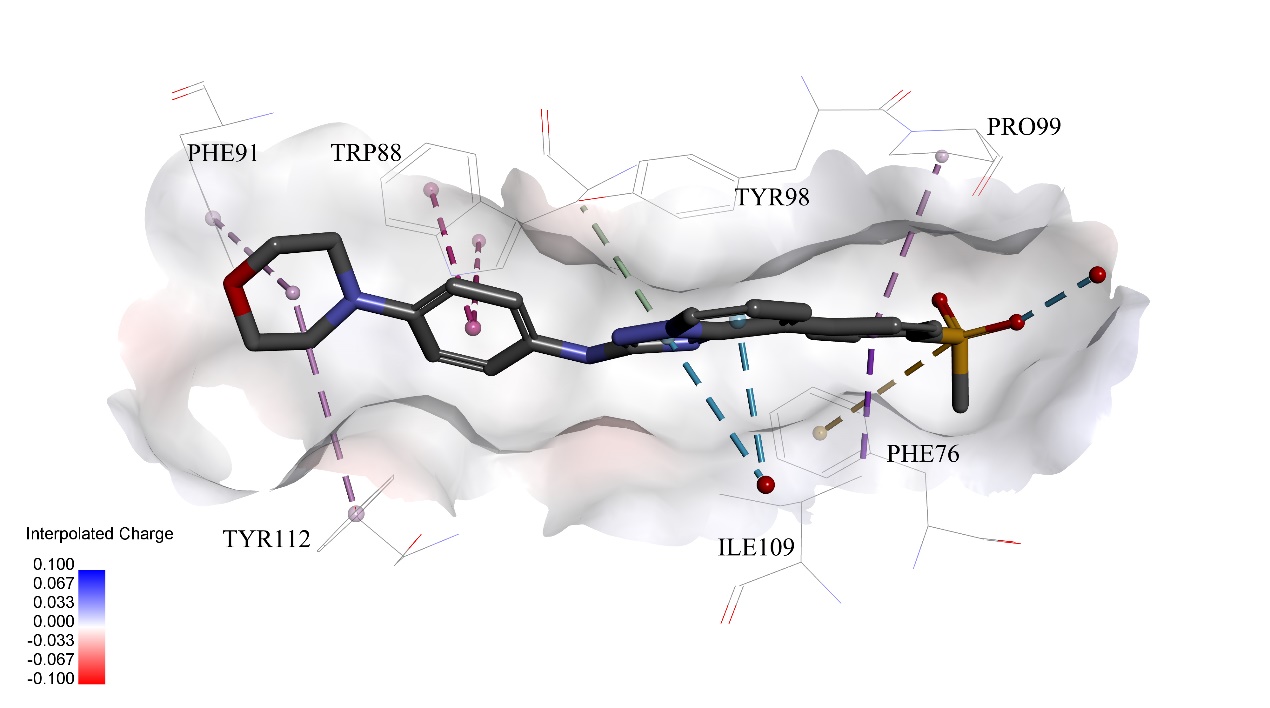


**Supporting Figure 11** Predicted binding modes of compound **2** to pVHL. The protein displayed as a gray surface and its key residues were shown with labels. All compounds are shown with only backbone atoms.





**Supporting Figure 12** the structure of **3**


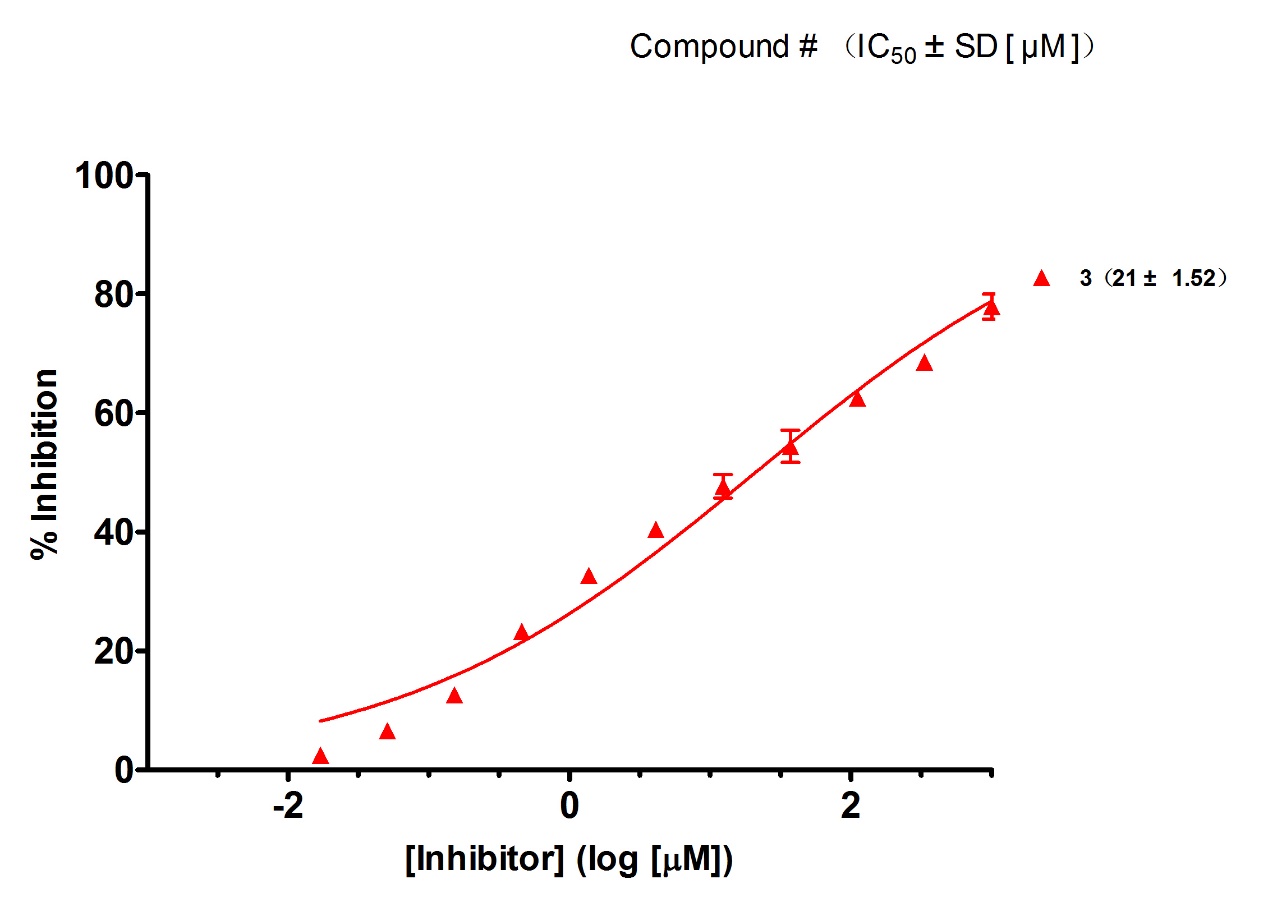


**Supporting Figure 13** the curve of inhibitory of **3**


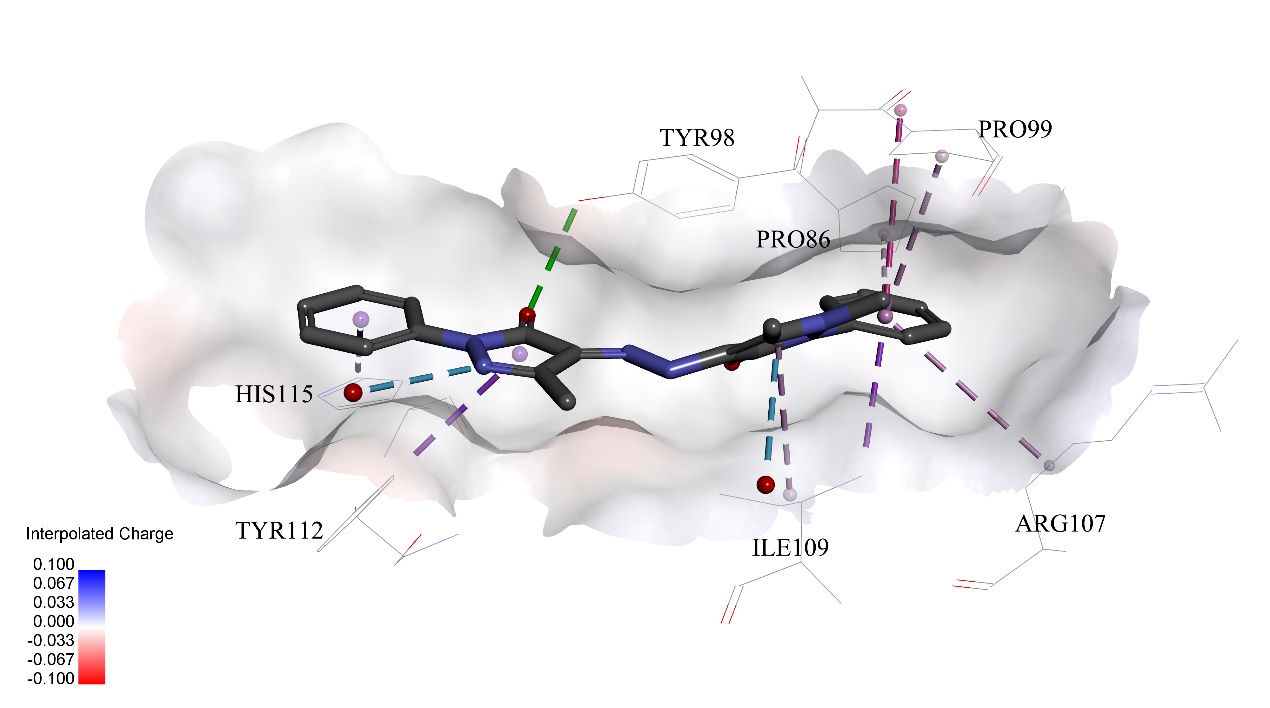


**Supporting Figure 14** Predicted binding modes of compound **3** to pVHL. The protein displayed as a gray surface and its key residues were shown with labels. All compounds are shown with only backbone atoms.





**Supporting Figure 15** the structure of **4**


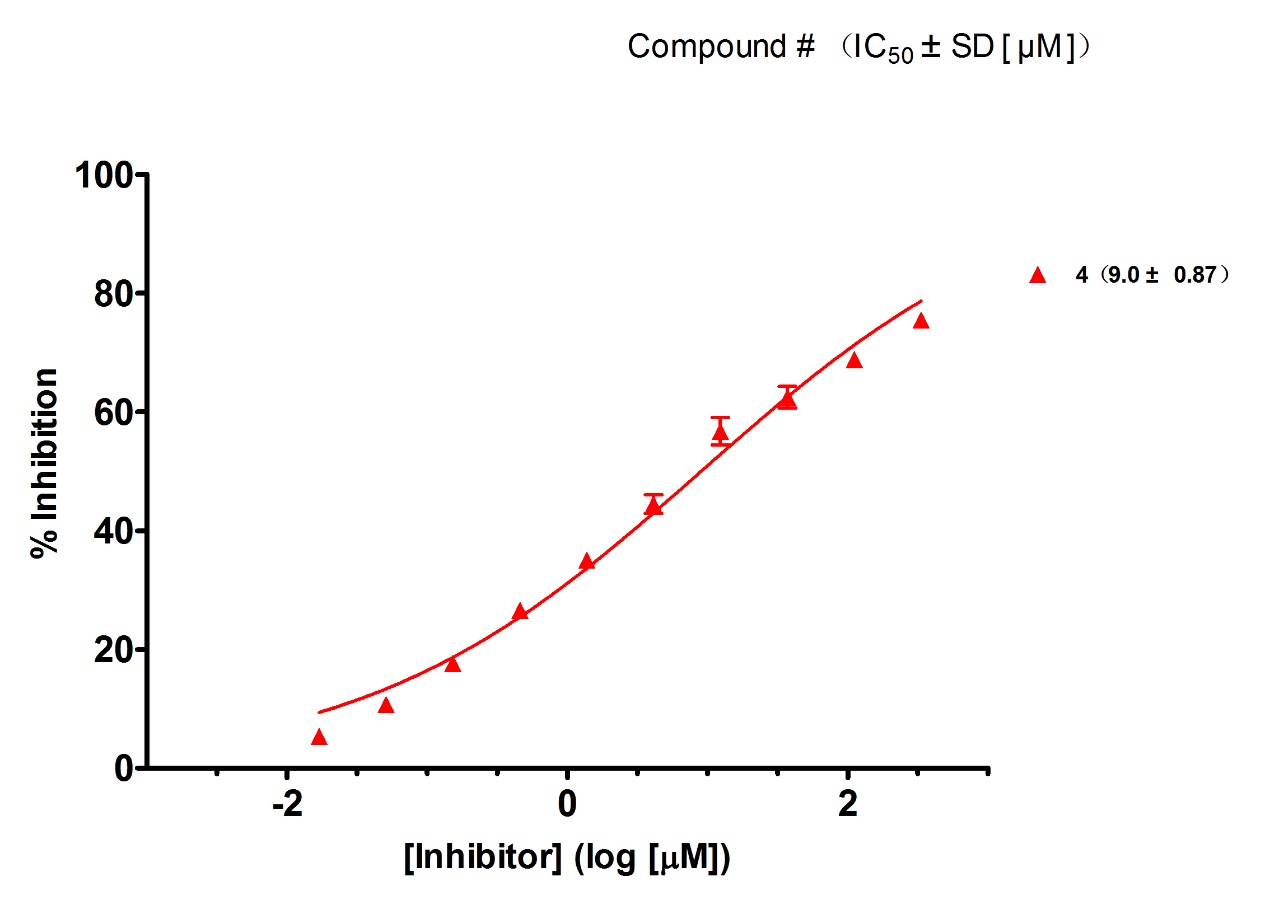


**Supporting Figure 16** the curve of inhibitory of **4**


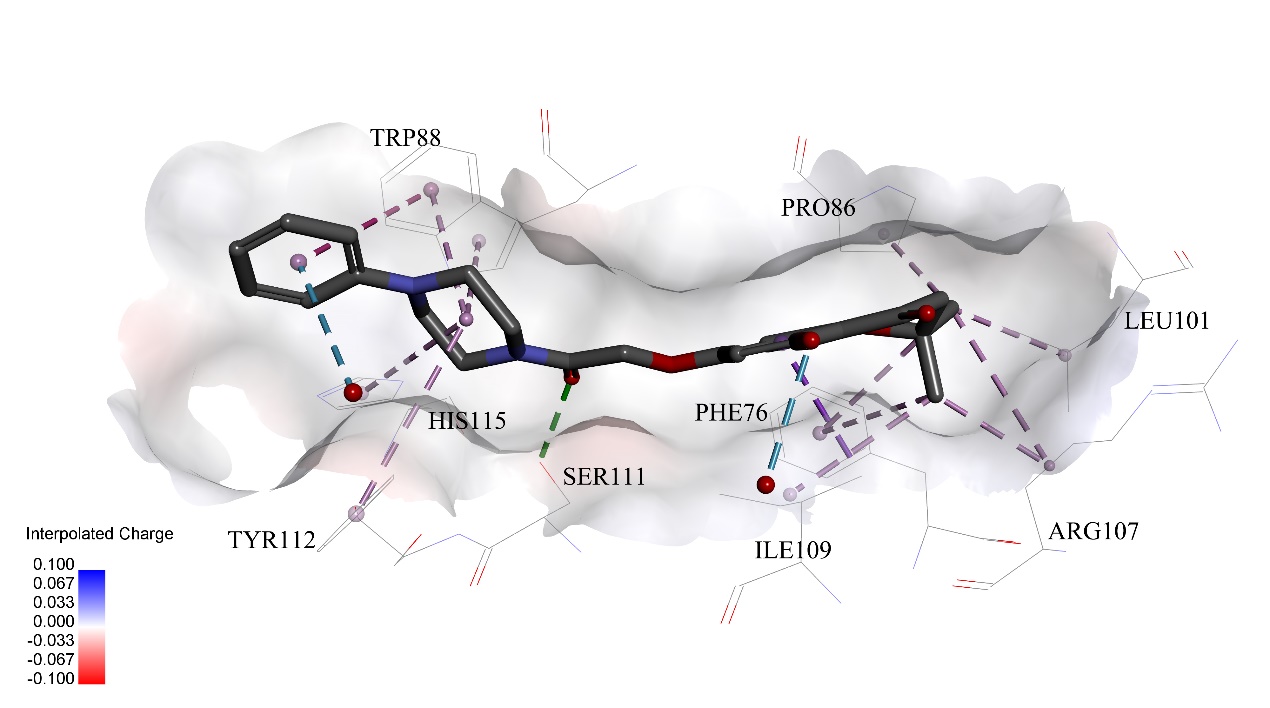


**Supporting Figure 17** Predicted binding modes of compound **4** to pVHL. The protein displayed as a gray surface and its key residues were shown with labels. All compounds are shown with only backbone atoms.





**Supporting Figure 18** the structure of **5**


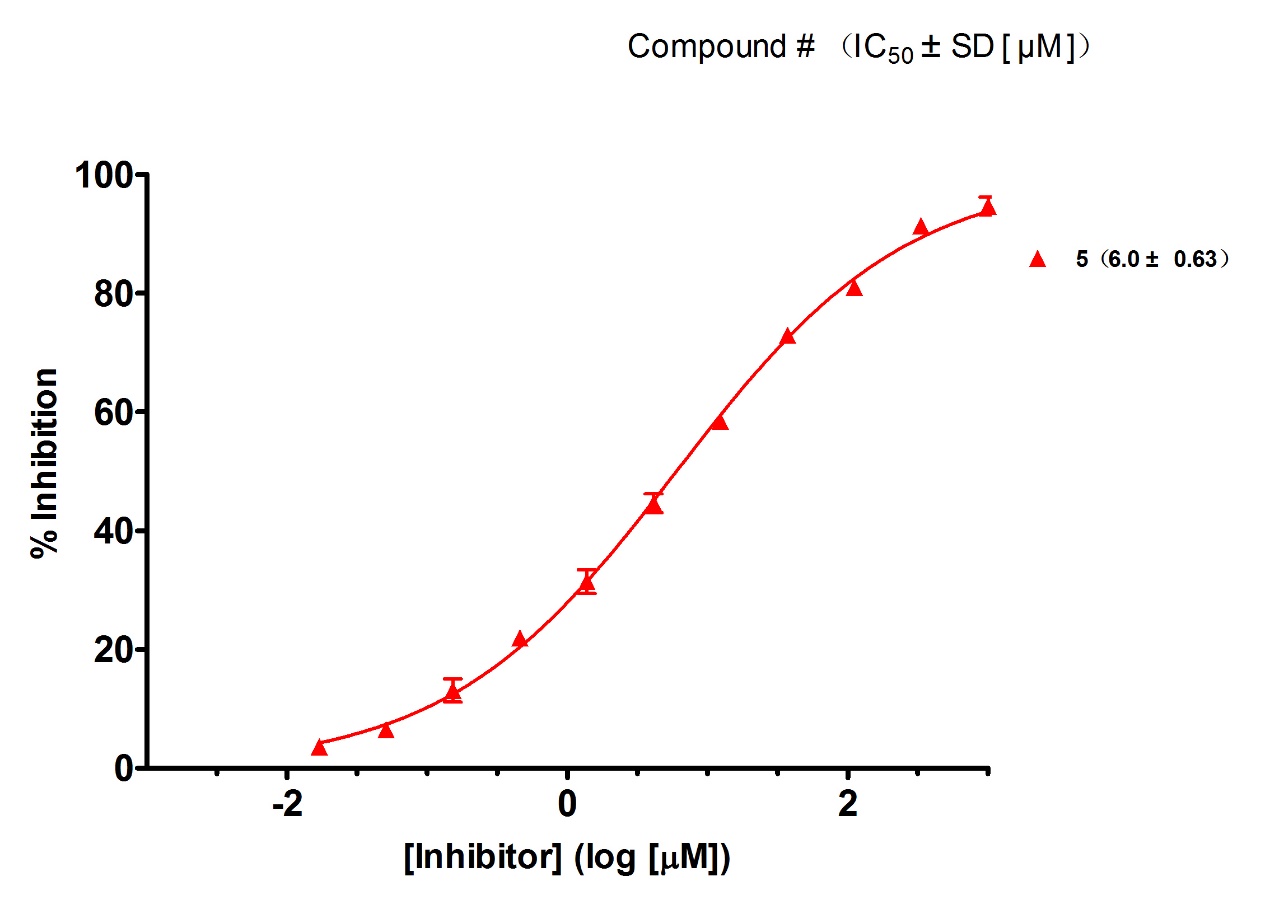


**Supporting Figure 19** the curve of inhibitory of **5**


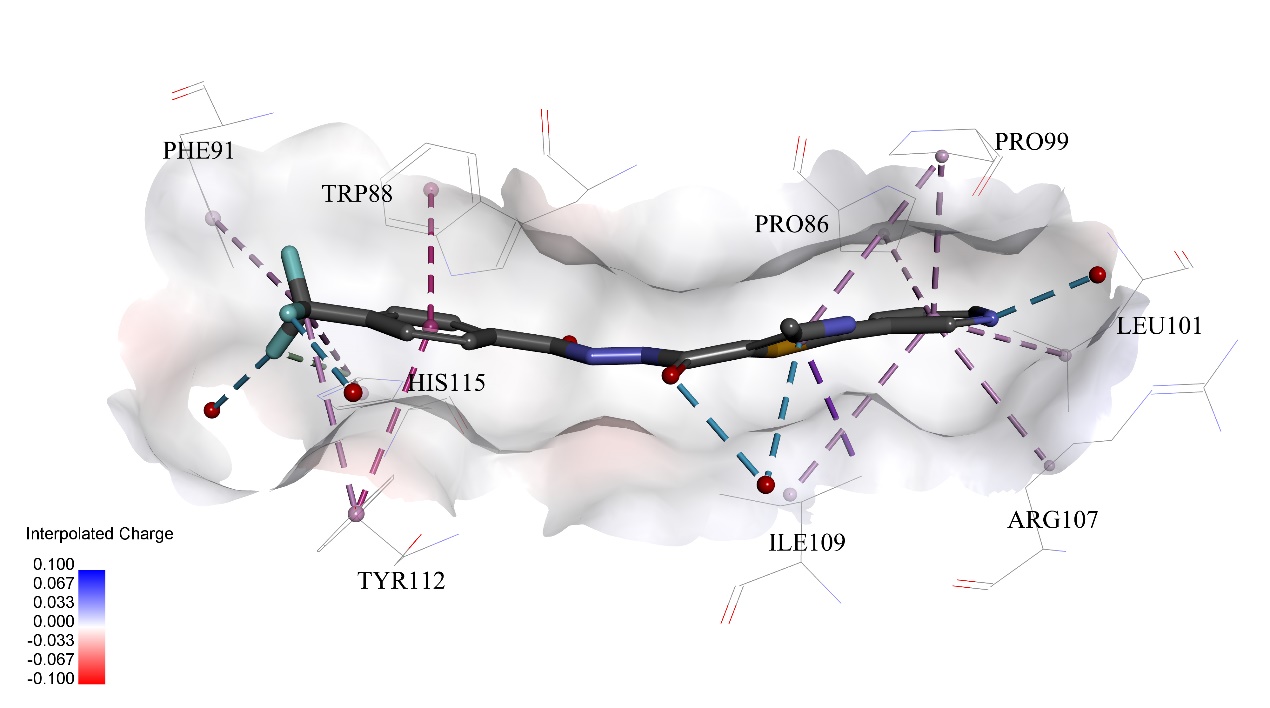


**Supporting Figure 20** Predicted binding modes of compound **5** to pVHL. The protein displayed as a gray surface and its key residues were shown with labels. All compounds are shown with only backbone atoms.





**Supporting Figure 21** the structure of **6**


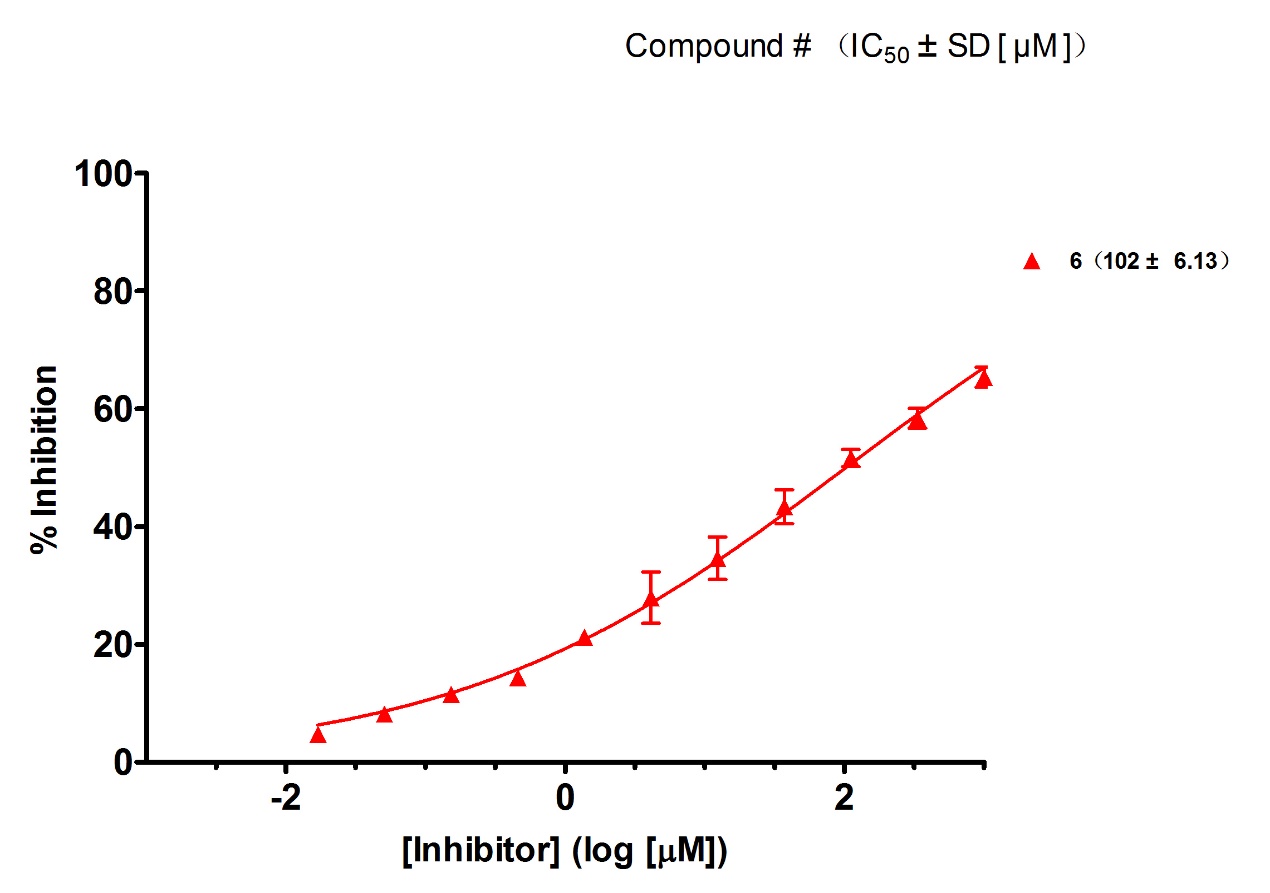


**Supporting Figure 22** the curve of inhibitory of **6**


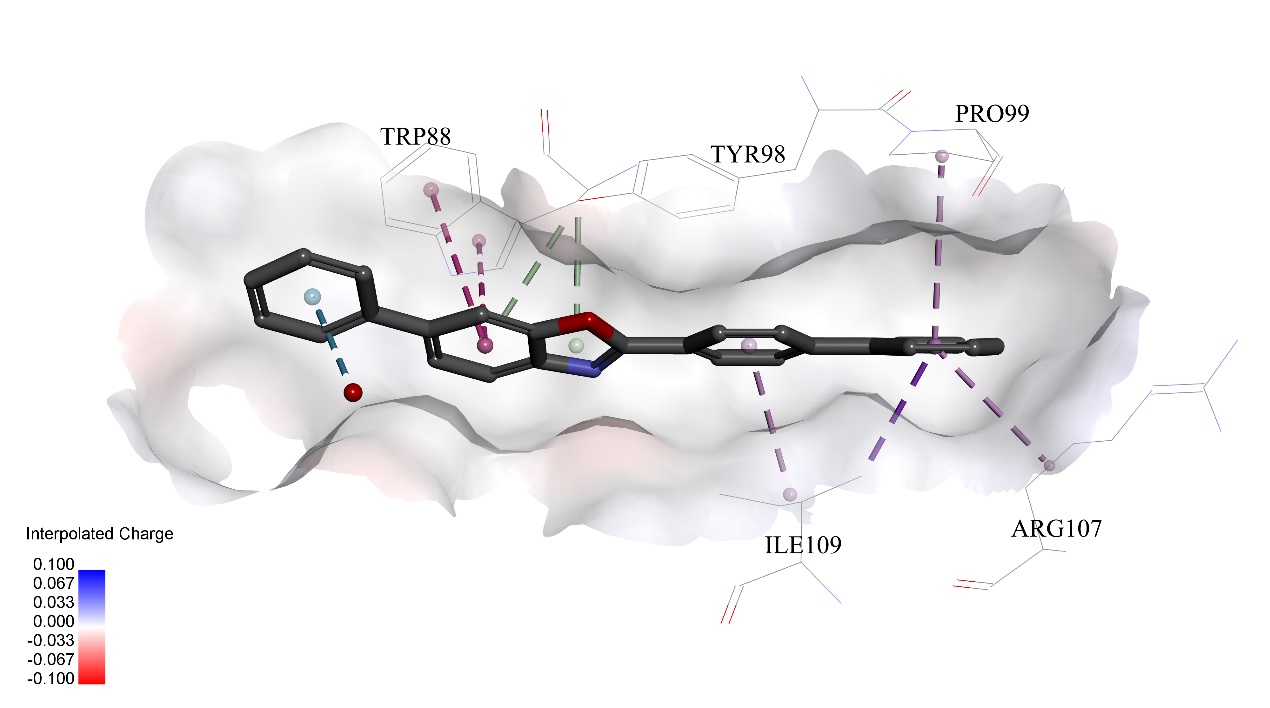


**Supporting Figure 23** Predicted binding modes of compound **6** to pVHL. The protein displayed as a gray surface and its key residues were shown with labels. All compounds are shown with only backbone atoms.





**Supporting Figure 24** the structure of **7**


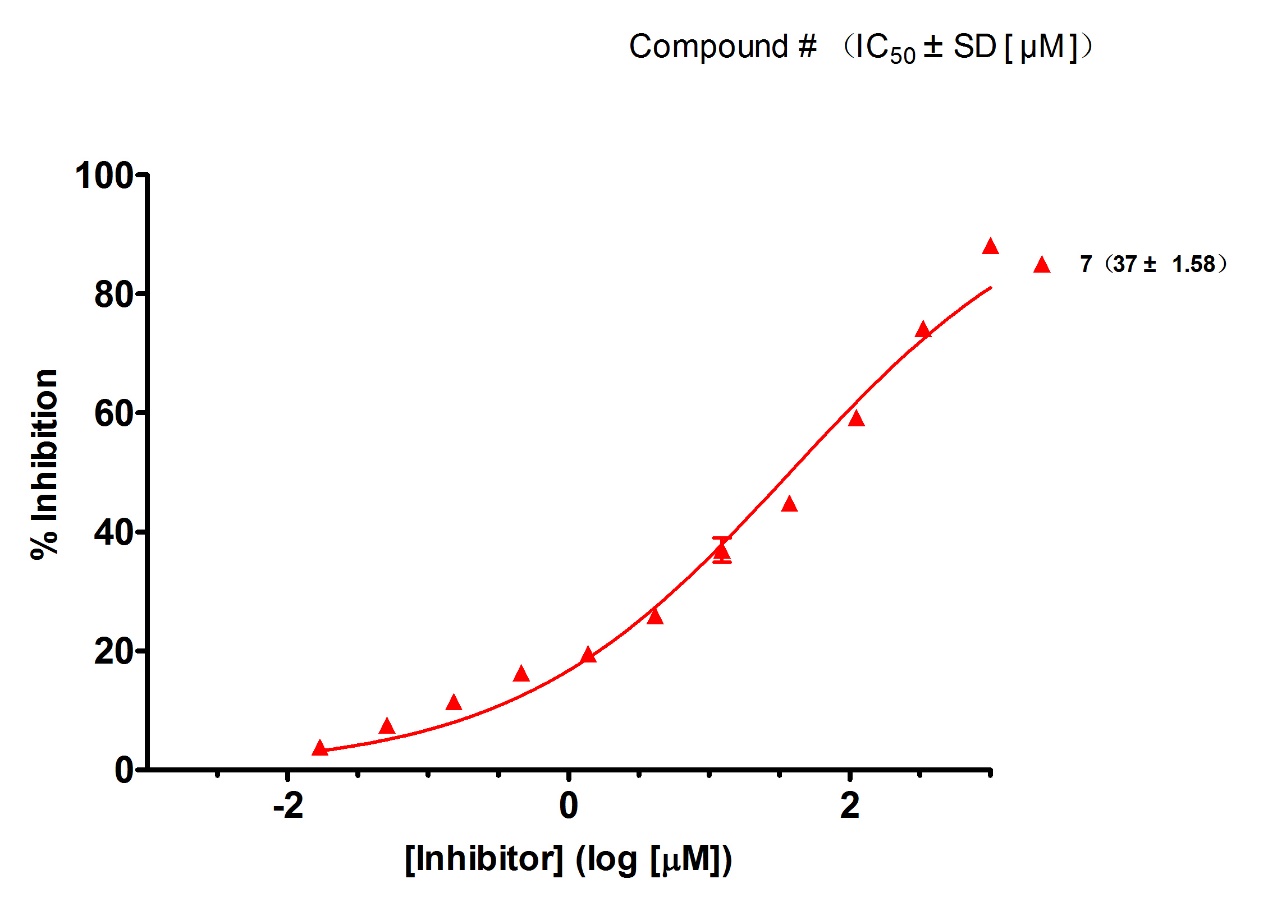


**Supporting Figure 25** the curve of inhibitory of **7**


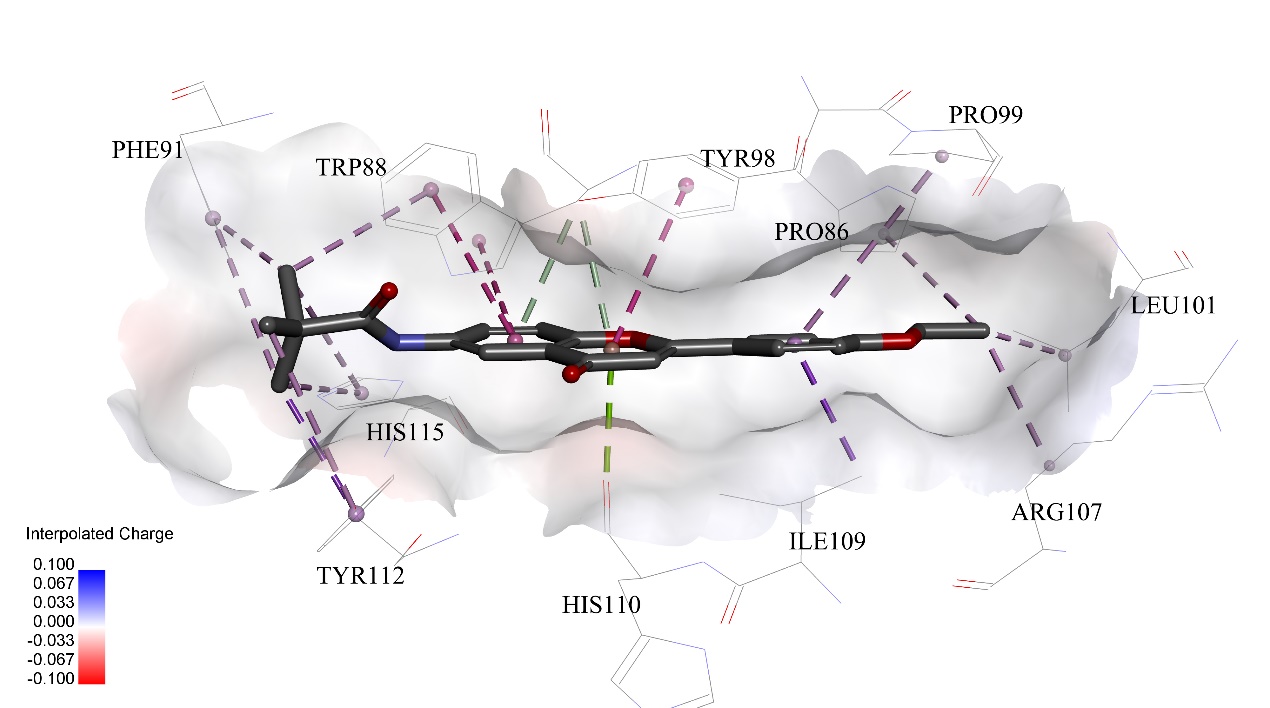


**Supporting Figure 26** Predicted binding modes of compound **7** to pVHL. The protein displayed as a gray surface and its key residues were shown with labels. All compounds are shown with only backbone atoms.





**Supporting Figure 27** the structure of **8**


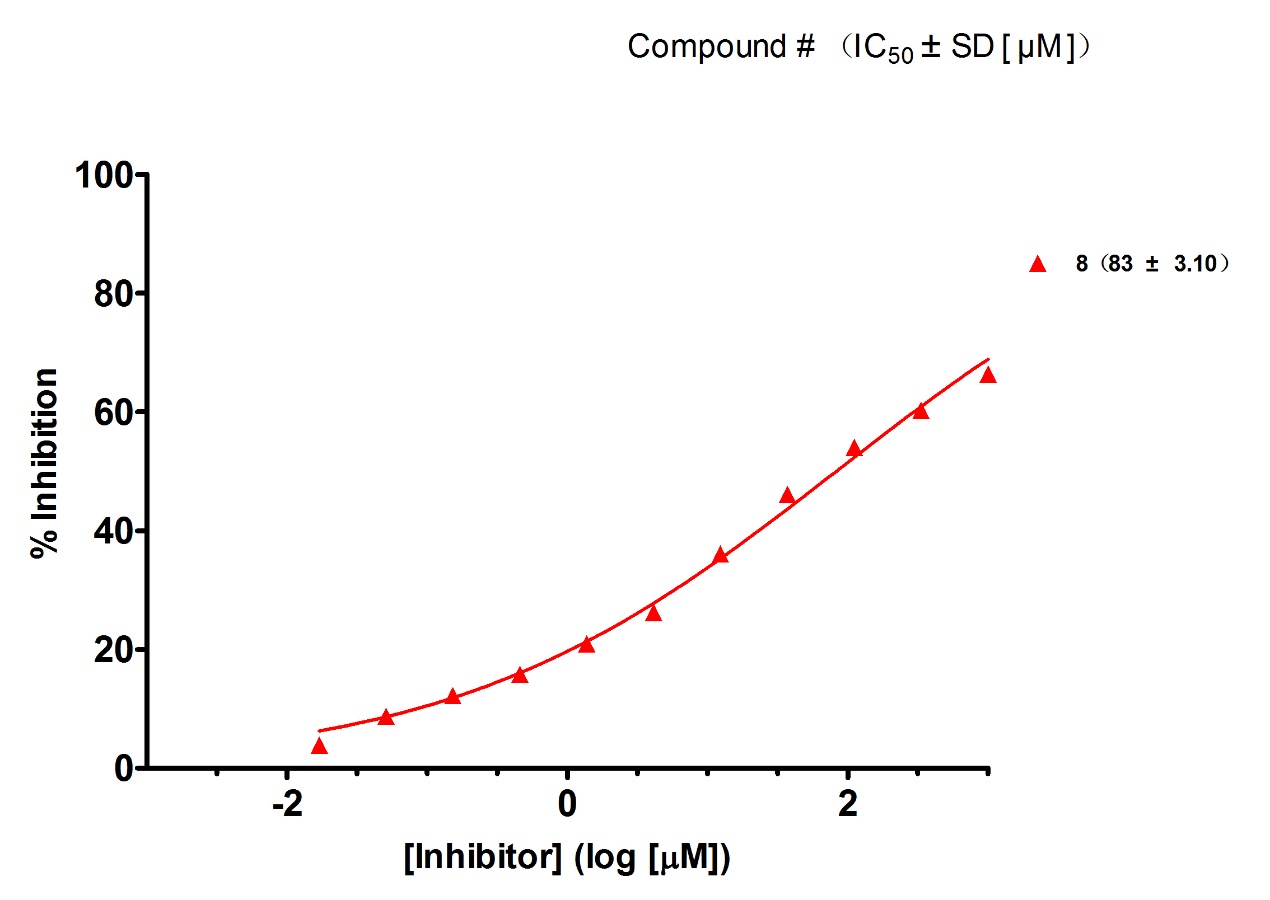


**Supporting Figure 28** the curve of inhibitory of **8**


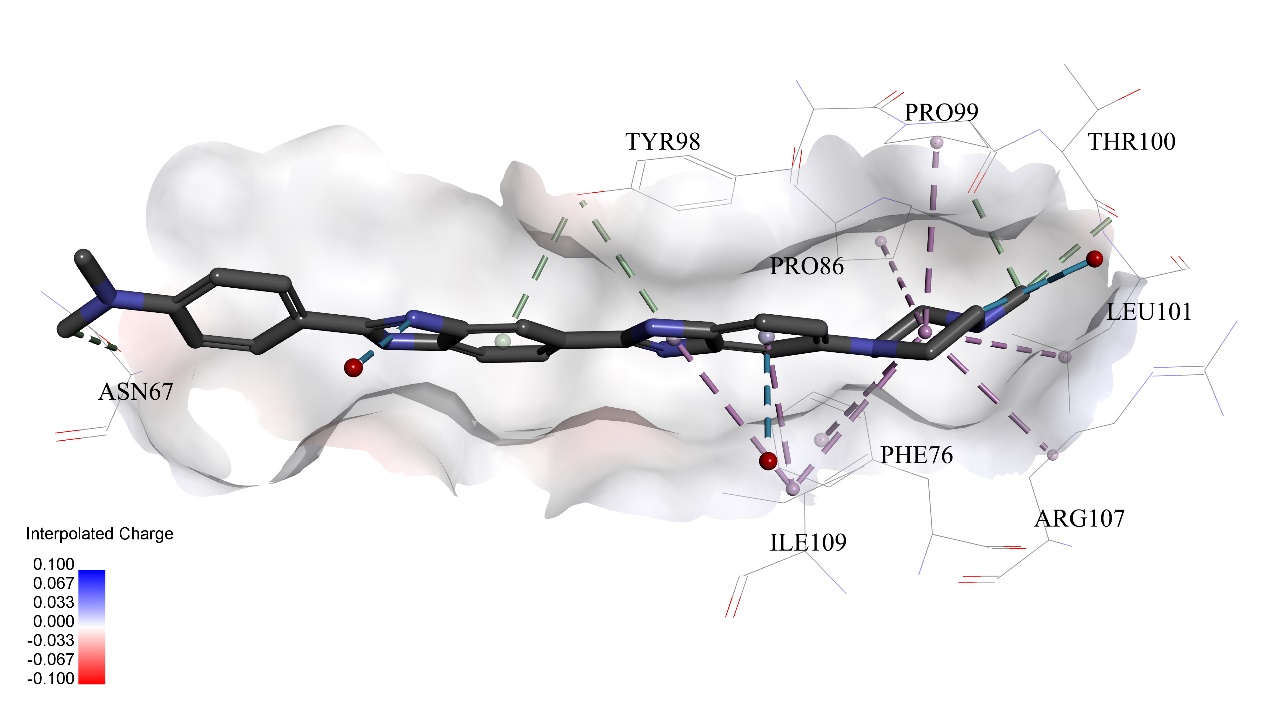


**Supporting Figure 29** Predicted binding modes of compound **8** to pVHL. The protein displayed as a gray surface and its key residues were shown with labels. All compounds are shown with only backbone atoms.





**Supporting Figure 30** the structure of **9**


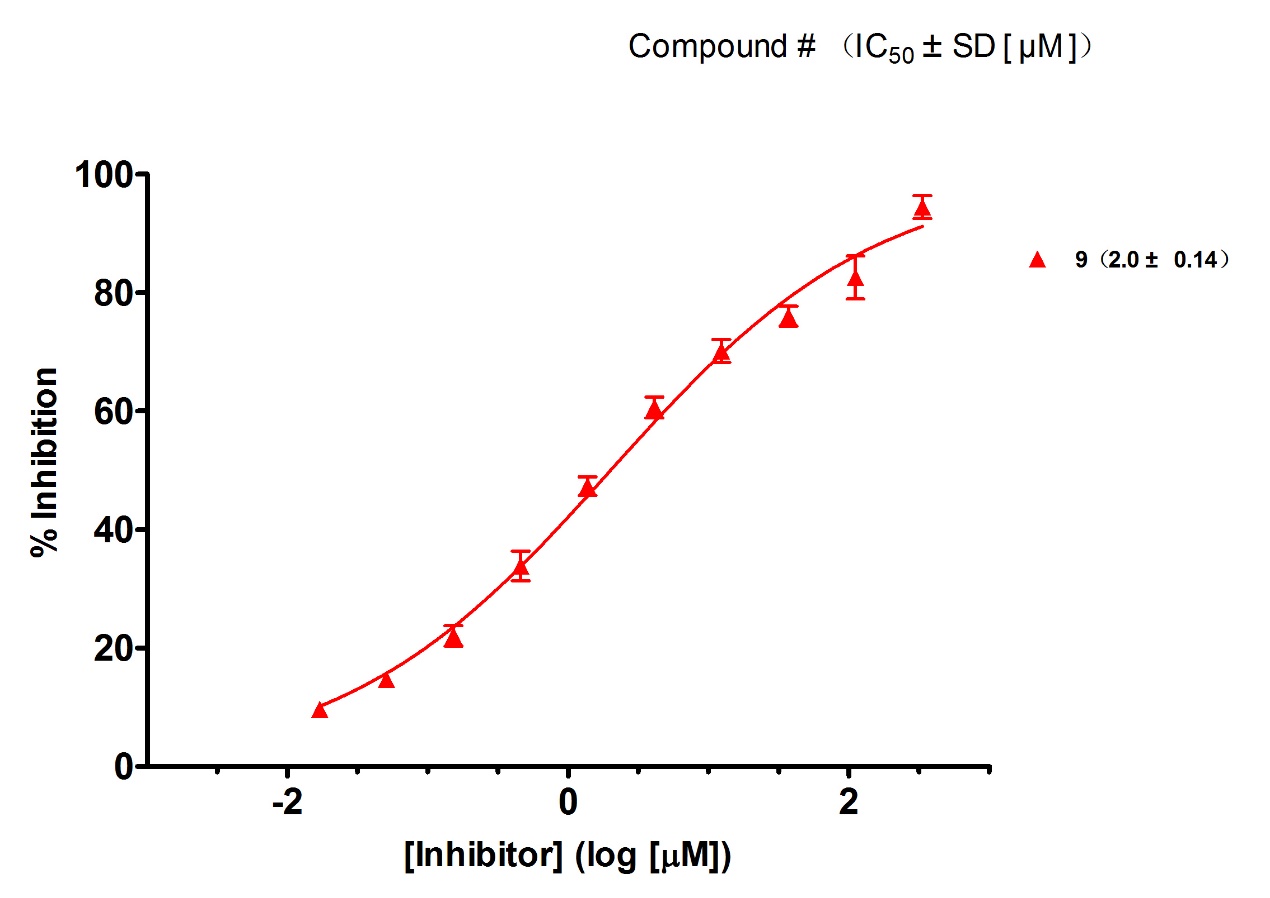


**Supporting Figure 31** the curve of inhibitory of **9**


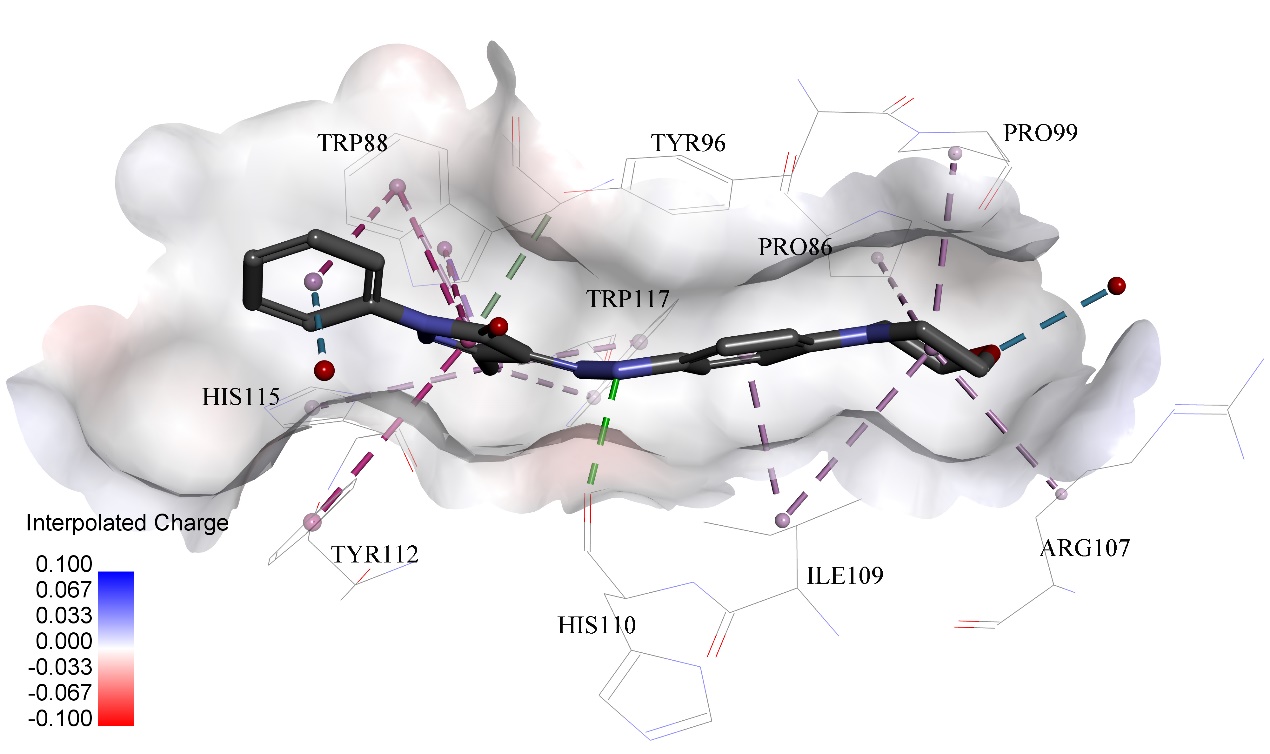


**Supporting Figure 32** Predicted binding modes of compound **9** to pVHL. The protein displayed as a gray surface and its key residues were shown with labels. All compounds are shown with only backbone atoms.
